# An all-atom protein generative model

**DOI:** 10.1073/pnas.2311500121

**Published:** 2024-06-25

**Authors:** Alexander E. Chu, Jinho Kim, Lucy Cheng, Gina El Nesr, Minkai Xu, Richard W. Shuai, Po-Ssu Huang

**Affiliations:** ^a^Biophysics Program, Stanford University, Stanford, CA 94305; ^b^Department of Bioengineering, Stanford University, Stanford, CA 94305; ^c^Department of Physics, Stanford University, Stanford, CA 94305; ^d^Aquarium Learning, San Francisco, CA 94117; ^e^Department of Computer Science, Stanford University, Stanford, CA 94305

**Keywords:** protein design, protein structure, generative modeling, sidechain generation, full-atom model

## Abstract

Proteins mediate their functions through chemical interactions; modeling these interactions, which are typically through sidechains, is an important need in protein design. However, constructing an all-atom generative model requires an appropriate scheme for managing the jointly continuous and discrete nature of proteins encoded in the structure and sequence. We describe an all-atom diffusion model of protein structure, Protpardelle, which represents all sidechain states at once as a “superposition” state; superpositions defining a protein are collapsed into individual residue types and conformations during sample generation. When combined with sequence design methods, our model is able to codesign all-atom protein structure and sequence. Generated proteins are of good quality under the typical quality, diversity, and novelty metrics, and sidechains reproduce the chemical features and behavior of natural proteins. Finally, we explore the potential of our model to conduct all-atom protein design and scaffold functional motifs in a backbone- and rotamer-free way.

## Significance

Proteins drive many biological processes; the ability to design and engineer their structure and function has potential for impact across science, medicine, and engineering. Generative modeling with deep neural networks has emerged as a powerful approach for modeling and controllably sampling from the distribution of protein structures. However, many methods ignore the sidechain atoms, which drive most of protein function, focusing only on the backbone conformation. We describe a structure and sequence codesign algorithm which can generate the full atomic structure of proteins across the diverse folds found in the PDB, offering a way to design proteins conditioned directly on functional elements of interest.

Structure, or more specifically the set of spatial chemical interactions proteins make with themselves and other molecules, remains the best rationalizable connection between protein sequence and function. Therefore, the ability to design proteins for novel functions often involves some modeling of structure, implicitly or otherwise. In order to have the most control over the arrangement of structural elements, it is often beneficial to design proteins de novo, specifying fully the structure and sequence of a protein from scratch (1, 2).

Within a protein structure, sidechains are the primary functional effectors and define both the intrinsic properties of the protein and the types of interactions a protein can make. While it is well known that the sequence (and therefore the set of sidechain) determines the global fold of the protein, the interactions of sidechains with other sidechains can also influence local packing and structure, as well as distal allosteric effects. Sidechains also mediate most unique protein functions, ranging from protein–protein interactions to enzyme catalysis and metal coordination. Their precise placement in designed proteins is essential for downstream function. Combined with the backbone atoms, the ability to model the full atomic protein structure is important for advancing protein design.

However, modeling sidechains in protein design is difficult: to specify which sidechains need to be modeled, the sequence must be known, at which point the protein is already fully determined. Thus, current de novo design methods rarely model the sidechains at all when specifying the sequence or structure, but instead sample the backbone structure and sequence separately, and then build the sidechains afterward (3–21). Some other structure-oriented design methods allow the sequence and structure to interact during the design process, allowing for codesign and for each representation to influence the other (12, 22–24). For some specific protein folds or classes such as antibodies, sequence and structure codesign has been explored, sometimes in an all-atom manner (25–29). While these methods have led to remarkable advances in our ability to design protein structures and sequences with increasing control, robustness, and usefulness, most have not yet deeply explored the generation of protein structures conditioned explicitly on all-atom structure or sidechains.

Here, we describe an all-atom protein generative model, Protpardelle, which codesigns the backbone, sequence, and sidechains of a protein together. Our method enables a key capacity in protein design: generating complete proteins with coherent sequences and all-atom structures. By codesigning the structure and sequence, the all-atom modeling approach allows the sequence to influence the backbone conformation through the sidechains, and vice versa. We accomplish this by developing a way to manage all of the sidechains at once during the generation process, which we dub “superposition” in reference to how quantum wavefunctions exist in multiple states before “collapsing” into a single state when observed. Model samples are of good quality, both in terms of consistency between structure and sequence as well as chemical fidelity of the sidechains. Preliminary exploration of design applications suggests that our model can be used to design new proteins in an all-atom context, as well as when conditioned on only the functional groups of protein sidechains. We also describe a performant backbone generative model as a special case of our model. Both models are computationally lightweight, which aids exploratory research; in service of this, we make our code available at https://github.com/ProteinDesignLab/protpardelle.

## Method

### A Simplified ODE for Protein Structure Modeling.

Diffusion or score-based generative models (30–33) have emerged as a powerful framework for generating high-quality data samples in continuous domains, including protein structures (17, 18, 20, 21, 34–36). They have shown promising results by utilizing an iterative generation mechanism that allows the model many opportunities to commit to and refine a sample. These methods are also highly amenable to conditioning, with several mechanisms to inject steering information and guidance; this is particularly relevant for protein design, since the final objective is almost always to produce a protein with some desired property (37–39). Due to these attractive properties, we use the diffusion paradigm to construct our generative model.

To review the basic approach of diffusion-based generative models, we can define [**1**] forward and [**2**] reverse SDEs that connect an interesting distribution (e.g., the data distribution, $p_{0}\left(x\right)$) to a tractable distribution (e.g., the isotropic Gaussian distribution, $p_{T}\left(x\right)$) (33). The forward SDE reduces the signal to noise ratio until data are destroyed to whitened noise, and the reverse SDE recovers realistic data from random initial noise by progressively denoising noisy data.

[1] $$\begin{array}{c} \;\text{d}x=f(x,t)\;\text{d}t+g(t)\;\text{d}w. \end{array}$$

[2] $$\begin{array}{c} \;\text{d}x=\left[f\left(x,t\right)-g{\left(t\right)}^{2}\nabla_{x}\log p_{t}\left(x\right)\right]\;\text{d}t+g\left(t\right)\;\text{d}w. \end{array}$$

Here, g(t) is a diffusion coefficient, w is the standard Wiener process, and f(x,t) is a drift term which is typically of the form f(t)x and describes a time-dependent scaling of the data. Different choices for the drift and diffusion coefficients give rise to the various variance-preserving and variance-exploding noise process formulations (31, 33). Given a score model that computes or approximates the score, or gradient of the log density of data $\nabla_{x}\log p\left(x\right)$, we can produce solutions to the reverse SDE by discretization and numerical integration, allowing us to generate data from noise. This score model is typically parameterized by a neural network trained with denoising score matching, which we call $D_{\theta }$ (40, 41).

In place of the reverse SDE, we can instead solve the probability flow ODE, which can be derived from the SDE using the Fokker–Planck equation and whose solution trajectories recover the same marginal distributions (33).

[3] $$\begin{array}{c} \;\text{d}x=\left[f\left(x,t\right)-\frac{1}{2}g{\left(t\right)}^{2}\nabla_{x}\log p_{t}\left(x\right)\right]\;\text{d}t. \end{array}$$

This enables a connection with continuous normalizing flows (CNFs) (42, 43) and thus a number of useful capabilities including a bijective map from data to latent representations via deterministic encoding and decoding, and exact likelihood computation. One particular configuration of this ODE does not scale the data and uses the identity function for σ(t)=t so that the noise level increases at the same rate as time, or diffusion progress (44).

[4] $$\begin{array}{c} \;\text{d}x=-t\nabla_{x}\log p_{t}\left(x\right)\;\text{d}t. \end{array}$$

**Fig. 1.**
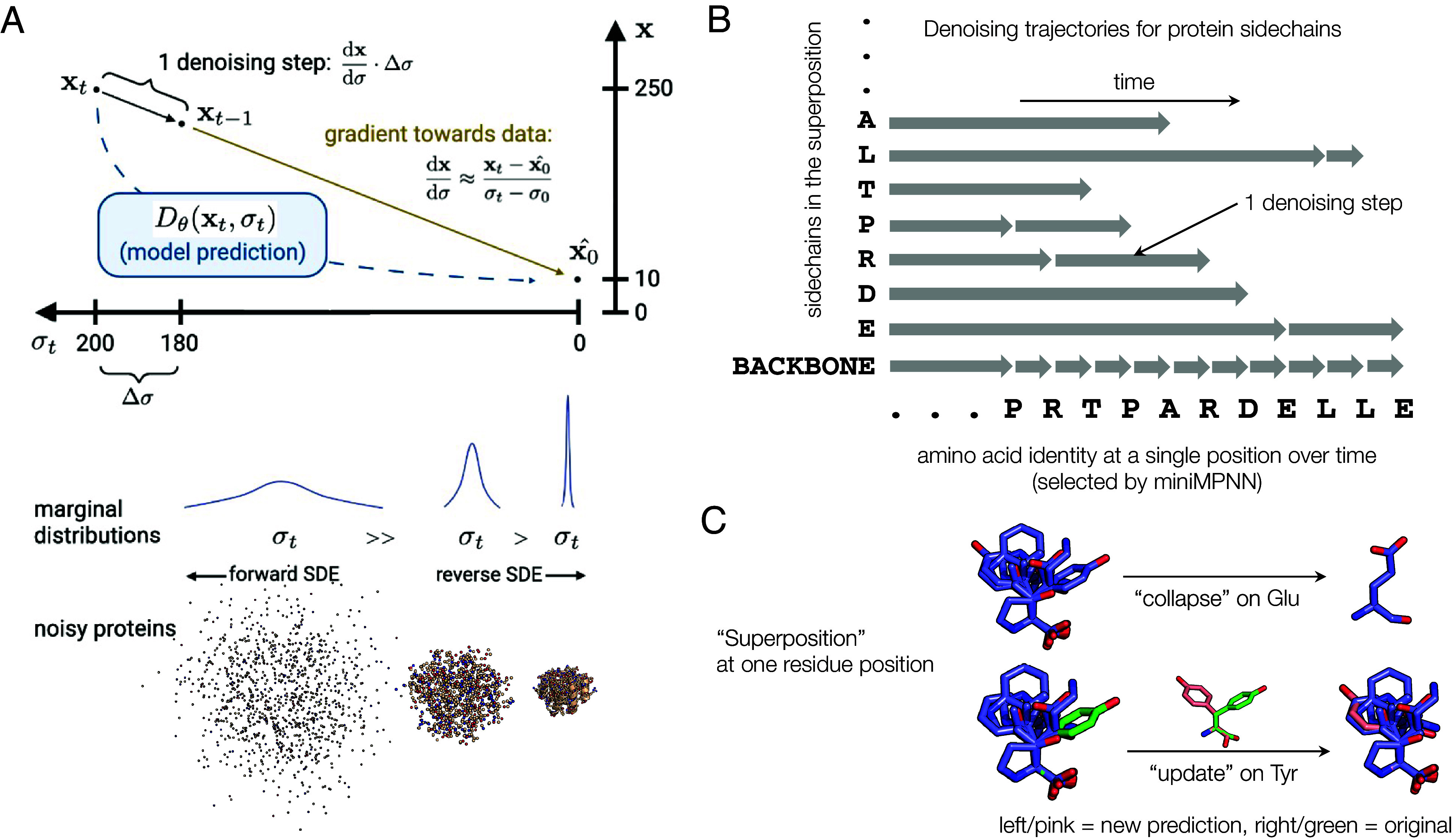
Superposition modeling approach and denoising scheme for Protpardelle. (*A*) The basic idea of denoising protein structures by integrating an ODE. Given noisy data $x_{t}$, we can run the denoising network to predict the fully denoised data, $x_{0}$. Given the quantities $x_{t}$, $x_{0}$, and the noise level $\sigma_{t}$, we can estimate the score, or gradient which points in the direction of data. We can then take a denoising step (integrating the ODE) by choosing a step size Δσ and computing an update Δx on $x_{t}$ which yields slightly denoised data $x_{t-1}$. We can repeat this many times to iteratively denoise our sample and produce protein samples. The noising process is defined by the marginal distributions, which noise protein structures by simply adding Gaussian noise to the atom coordinates. The scale of these Gaussians increases linearly with time, which induces mostly linear ODE solution trajectories. In our model, the forward noise process acts only on real proteins (with one sidechain per amino acid), whereas the reverse denoising process acts on the full superposition over all possible sidechains. (*B*) A visualization of the Protpardelle sampling routine for a single residue position. The vertical axis lists the structural elements being denoised (i.e., the atoms of the 20 sidechains in the superposition, plus the backbone atoms). The horizontal axis denotes progression in sampling time, with each amino acid denoting the amino acid predicted for this position at a given timestep. Note that this amino acid prediction can change from step to step. Briefly, at each timestep, we use the predicted amino acid to collapse the superposition and form a “real” but noisy protein, predict denoised positions for each of the atoms in this protein, and then take a denoising step for selected atoms. The size of the denoising step for each atom or sidechain is determined by the last time that atom or sidechain took a denoising step. Each amino acid sidechain from the superposition is denoised only when it is selected by the sequence model. This means that the size of the denoising/integration step varies depending on how frequently that amino acid is predicted. The backbone is denoised at every step since these atoms are common to all amino acids. For more details and the actual sampling algorithm, see *Method* and Algorithm 1. (*C*) An example visualization of the sidechain superposition idea and how it might be collapsed or updated, functions which at each denoising step. Sidechains for all 20 amino acids are modeled at once, shown here aligned on the N, CA, and C atoms for a single residue position. Given an amino acid type, we can collapse the superposition from all states to a single state, which yields a “valid” residue or protein. Alternatively, given an amino acid type and newly predicted coordinates for that sidechain, we can update the superposition with new information.

The marginals associated with this ODE are $p_{t}\left(x\right)=N\left(x,\sigma_{t}^{2}\right)$, which can be interpreted as adding or removing Gaussian noise of constantly increasing scale during forward and reverse diffusion. This ODE structure was originally motivated to produce more linear solution trajectories with reduced truncation error (44, 45) and can also be understood as the linearly interpolating optimal transport (OT) map sending the initial distribution $p_{\sigma_{\min}}\left(x\right)\approx p_{0}\left(x\right)$ to the prior $p_{\sigma_{\max}}\left(x\right)\approx N\left(0,\sigma_{T}^{2}\right)$ (46, 47). As such, it facilitates straighter ODE solution trajectories in a manner similar to OT flow matching approaches (48, 49). Intuitively, integrating this ODE amounts to approximating the score with an estimate of the ground truth and then taking a small step Δt or Δσ in this direction (Fig. 1*A*). (Since σ:=t under this configuration, we will abuse our notation and use σ, t, and $\sigma_{t}$ somewhat interchangeably to indicate the noise level or progress in the diffusion process. Occasionally, we will use t−1 to indicate the succeeding timestep during sampling even though our model is defined on continuous time.) We will use $x_{0}$ to denote a sample from $p_{0}\left(x\right)$, i.e., unnoised data, and $x_{t}$ to denote samples from the marginal distributions $p_{t}\left(x\right)$, i.e., noised data.

Protein structure data can be handled in many different ways; one simple and descriptive approach is to treat it as a point cloud in 3D space. To apply this ODE to noise protein structures, we would add Gaussian noise with variance $\sigma_{t}^{2}$ independently and identically to the x, y, and z coordinates of each atom in the structure (backbone or sidechain). As physical objects in 3D space, the atomic coordinates of protein structures obey the symmetries of rigid body rotation and translation (but not reflection). Thus, our noising process should also consider the same symmetries. We note that the isotropic Gaussian distribution in n dimensions is symmetric and thus invariant to rotations, yielding an SO(n)-invariant density. Additionally, we always move the center of mass for all protein structures to the origin, which further ensures that the added noise is invariant to translation, yielding an SE(n)-invariant density (21). This noise distribution induces an SE(n)-equivariant diffusion process (50), where the noisy protein structures $x_{t}$ remain centered at the origin while rotating together with the original, true protein structure $x_{0}$.


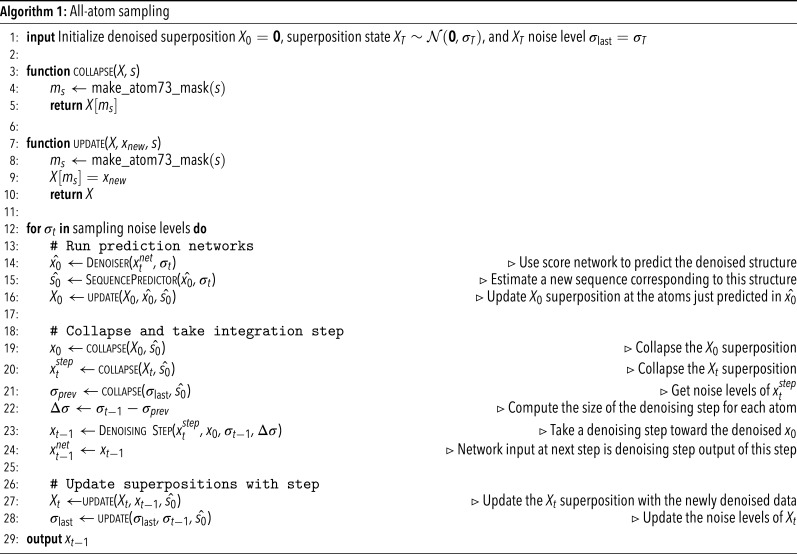


### Sampling with an All-Atom Superposition.

Training a score network to denoise protein backbone atoms (the N, CA, C, and O atoms) is straightforward, and we will also discuss our results with a backbone-only generative model. However, all-atom protein modeling presents an interesting challenge not only because of the dual continuous and discrete nature (structure and sequence) of proteins but also because the discrete sequence directly defines which atoms are present in the 3D structure. For example, serine has five heavy atoms, N, CA, C, O, CB, and OG, whereas histidine has 10 atoms, N, CA, C, O, CB, CG, CD2, ND1, CE1, and NE2. Five of these atoms (N, CA, C, O, and CB) are common to both amino acids, but serine has an atom missing in histidine, and histidine has five atoms missing in serine. This creates a chicken-and-egg problem: for each position, we cannot know which sidechain atoms to build without knowing the amino acid identity, but if we know all the amino acid identities, then the protein is already specified [Barring posttranslational modifications and other biological processes, proteins are entirely determined by their sequence (51)].

Nearly all current generative modeling paradigms utilize deep neural networks which work with fixed-size input and output, with length and shape differences between unique data points handled by masking. With few exceptions (52), all such models first fix the dimensionality of the data and then simulate generative processes conditioned on this dimensionality. For all-atom protein generation where both the protein structure and its sequence are unknown at the beginning of sampling, the estimated sequence evolves with time and therefore the structure consists of different atoms at each time step. Practically speaking, this means that not only does the data change due to the noise process, but the dimensionality and mask itself also change with each diffusion timestep. This remains the case whether sidechains are represented as sets of atoms or as sequences of chi angles. This makes it difficult to define a diffusion process which transforms data smoothly with time, if the dimensionality is changing and data are disappearing and reappearing at each step.

To address this challenge, we define our denoising process to act on a superposition of protein structure states—that is, the protein backbone and the coordinates of each of the twenty possible sidechains at once. This is clearly an unrealistic model of protein structure but allows us to handle the uncertainty associated with changes to the sequence in time. Given a sequence, we can “collapse” this superposition by selecting the sidechain states that correspond to this sequence to yield an all-atom protein structure. During structure generation, we maintain both an estimate of the fully denoised superposition state ($X_{0}$) and the current noisy state ($X_{t}$). At each denoising step, we collapse the $X_{t}$ superposition to produce a single noisy protein structure $x_{t}$ which we can use to predict the denoised data $x_{0}$ with the score network. This predicted $x_{0}$ can be used to update our $X_{0}$ estimate and to predict a new sequence. Then, the actual denoising step occurs: We collapse $X_{t}$ and $X_{0}$*using the new sequence* to get $x_{t}$ and $x_{0}$, and then we integrate the ODE using these two quantities (see Algorithm 1 and *SI Appendix*, S1 for the full pseudocode). We use notations $x_{t}^{\mathrm{net}}$ and $x_{t}^{\mathrm{step}}$ to distinguish the network input $x_{t}$ and the denoising step input $x_{t}$, respectively: Note that the score network prediction and the denoising step are decoupled in our method. The output of the denoising step $x_{t-1}$ at timestep t is exactly the $x_{t-1}^{\mathrm{net}}$ at the following timestep t−1; this arrangement is so that inputs to the network are always at the same noise level in the ODE solution trajectory. This superposition approach can be viewed as a form of expectation maximization, with an E-step for inferring the sequence variables and an M-step for gradient steps on the data likelihood (*SI Appendix*, section A).

A key insight of our approach is that the integration step size can vary for different atoms, and the ODE discretization need not be identical for all atoms (Fig. 1*B*). In this approach, the backbone atoms (N, CA, C, and O) are denoised at every iteration of the algorithm, and the various sidechain atoms are denoised only when the sequence design model selects the corresponding amino acid for that position. Thus, the sidechain atoms will typically see larger Δσ integration steps than the backbone at any point in time. Mechanically, the superpositions are stored in an “atom73” representation which indexes the N, CA, C, CB, and O atoms and then each amino acid’s sidechains independently. The collapse and update functions are mask-based interactions with the atom73 representation (Fig. 1*C*). To sample, we adapt a modified version of the stochastic sampling routine outlined in Karras et al. (44), which offers a high degree of flexibility to customize the sampling process. This routine uses the Euler method to integrate the ODE while injecting noise at each step [similar to Langevin dynamics or predictor–corrector methods (32, 33)] and accepts several tunable hyperparameters which we find can have a large effect on sample quality. The most impactful of these are the number of denoising steps, the amount of stochasticity added at each step (*s_churn*), and a scale applied to the denoising step (*step_scale*) (*SI Appendix*, Tables S1 and S2). Coarsely, the effect of scaling the score with some scalar β can be seen by rewriting $\beta \cdot \nabla_{x}\log p_{t}\left(x\right)$ as $\nabla_{x}\log p_{t}{\left(x\right)}^{\beta }$. Thus, the step scale can be interpreted as an inverse temperature parameter which sharpens the distribution and redistributes density from the tails to the modes.

### Training the Score Network.

Despite the fact that the model must manage all possible sidechain positions at once during the reverse process, the forward process does not require any superposition modeling at all, because the score is only predicted on the collapsed states during sampling. This means that we can noise real data to generate training examples, and the score network can be trained directly on these examples. This simplifies the training scheme significantly and enables many optimizations and experiments to occur cheaply at inference time.

Inputs to the model are samples from the marginal distributions $x_{t}∼p_{t}\left(x\right)=N\left(x,\sigma_{t}^{2}\right)$, which can also be written as $x_{t}=x+z;z∼N\left(0,\sigma_{t}^{2}\right)$ to highlight how noise is added to the data. In contrast to other protein diffusion models which find it necessary to add auxiliary domain-specific loss terms, we use only a single denoising score matching loss, with loss weighting:

[5] $$\begin{array}{c} E_{x_{0}∼p_{0},t∼p_{\mathrm{train}}}\left[\lambda \left(\sigma \right)\|D_{\theta }\left(x_{t},\sigma_{t}\right)-x_{0}{\|}_{2}^{2}\right]. \end{array}$$

The score network $D_{\theta }$ is a simple U-ViT from computer vision which we augment with network preconditioning, a scaling scheme to streamline the training objective (44, 53). In essence, inputs and outputs of the network are scaled and interpolated so that inputs are of consistent variance across training examples and noise levels. The loss weighting is determined by this preconditioning. Noise levels used to corrupt data were sampled from a log-normal distribution rather than the usual uniform distribution, which can be viewed as enriching the dataset at noise levels which are most critical for perceptual sample quality (44). Compared to other approaches which alter the loss weighting at different noise levels, this strategy offers a training objective with lower variance. For more details on training, see *SI Appendix*, sections B and C.

We note that the loss function and network architecture are not equivariant to transformations in SE(3). Equivariance confers a useful inductive bias which in a generative regime mostly serves to improve sample efficiency; we find that when trained with the appropriate data augmentations (i.e., random rotations), the model remains highly performant (*SI Appendix*, section C). In every use case of the network, the translational frame is canonicalized by centering at the origin and the rotational frame is implicitly provided as conditioning to the model via the inputs, which include the noisy data or fixed inputs for inpainting or both. Since the diffusion process is based on SO(3)-invariant densities, converting the model to a fully SE(3)-equivariant one is only a matter of replacing the U-ViT module with an equivariant module which operates on 3D coordinates. Such equivariant networks are typically more computationally intensive; we opted for the relative compute efficiency of nonequivariant networks and find significant benefits in this area. Our backbone model is one to two orders of magnitude faster than similar equivariant models, training in 4 GPU-days and generating structures at a rate of 0.1 to 0.5 s per 100 residues, compared to 2 to 24+ GPU-weeks of training and 20 to 60 s per 100 residues in sampling for other backbone diffusion models (17, 18, 21).

### Sequence Codesign.

An all-atom generation approach also necessitates a way to estimate the correct sequence at each step of generation. In practice, any (fast) predictor of protein sequence given structure can fill this role. For this, we used the ProteinMPNN graph neural network architecture which has been shown to capture an efficient locality-based inductive bias and produces strong sequence design results when used to parameterize an autoregressive model (54). We adapted the architecture to produce a “mini-MPNN” model by removing the causal (autoregressive) mask which improves sampling time complexity significantly from O(N) to O(1) and augmenting the intermediate MLP layers with noise conditioning, allowing it to be trained on higher noise levels (55). As input to the network we provide the denoised $x_{0}$ structure and optionally the predicted sequence from the previous step, a strategy akin to self-conditioning (56). With this approach, the sequence estimate becomes more and more accurate as the structure becomes better defined, so the “correct” sidechains are denoised more frequently as the trajectory progresses. We note that we do not define a diffusion process on the protein sequence; we only codesign the sequence with the structure.

It is possible for the sequence to influence the structure in two ways. One is the intended behavior where the positions of sidechains in space induce changes to the backbone to accommodate these sidechains. The second is for the model to infer the sequence from the atom mask and memorize the structure given this sequence, or *sequence leakage*. This creates a distribution shift issue during sampling where if the sequence is not plausible, the network is asked to denoise unusual inputs and struggles to produce valid structures. We remedy this by obscuring the atom mask by noising all 37 unique atom position inputs instead of only the atoms corresponding to the sequence. Later in the diffusion process as the structure information becomes clearer, the sequence predictions also improve and the problem recedes. Further discussion of this is given in *SI Appendix*, section D.

## Results and Discussion

### Developing a Method for All-Atom Generation.

We first sought to establish the feasibility of the general ODE and denoising scheme by training the model on protein backbones only, i.e., generating the N, CA, C, and O atoms. We made a number of architectural and training improvements which we found to improve performance (see *SI Appendix*, section E for more details). One notable feature of diffusion models relative to other types of generative models is inference-time flexibility. We found that significant gains in sample quality can be obtained by tuning sampling hyperparameters, which is inexpensive (*SI Appendix*, Table S1). In particular, tuning the step scale and the level of churn during the sampling process has a major effect on sample quality (*SI Appendix*, Tables S1 and S3).

With a baseline structure generation model in place, we next explored the capacity of the miniMPNN model to codesign the sequence during the structure diffusion. Following similar training procedures as for the original model, we found that miniMPNN was adequate as a structure-conditioned sequence predictor, achieving ∼38% sequence recovery on a validation set and a mean scRMSD of ∼8 in 1 to 5 sampling steps. We found that the base performance of the model as well as the many design steps (100 s) used for structure diffusion led to lower-quality sequences. To resolve this issue, we replaced the sequence prediction at the final step with a prediction from the full pretrained ProteinMPNN (54). This improved the sequence estimate and thus the self-consistency of the designed proteins. However, the fast prediction of coherent sequences with miniMPNN was still needed during the sampling trajectory—full ProteinMPNN was too slow, but randomly selecting sequences also resulted in much lower-quality samples due to the sequence leakage issue (see *Sequence Codesign* and *SI Appendix*, section D).

With a basic approach in place for structure and sequence codesign, to enable all-atom protein generation, we needed to also diffuse the sidechains. The simplicity of our training scheme made this very straightforward: since our forward diffusion process is identical for each atom, we simply increased the number of atoms per residue. The model was able to generalize to this extension, though with slightly weaker denoising performance (*SI Appendix*, Fig. S1), and we pursued a few strategies to improve the quality of sampled sidechains (*SI Appendix*, section E). In particular, we found that running a second sampling stage conditioned on both the backbone and sequence (i.e., essentially conducting rotamer packing) entirely removed any faulty sidechains. This two-stage sampling strategy added very little sampling time since it did not involve running miniMPNN or ProteinMPNN. Together, these modifications and improvements allowed us to consistently generate high-quality all-atom protein structures.

### Evaluating the Generative Model.

We evaluated our model on three main properties which are relevant for generative models, all related to sampling: the quality (broadly defined), diversity, and novelty of model samples (*SI Appendix*, section F). Intuitively, we desire our model to generate “good” samples that exhibit structural diversity and can generalize beyond the training set. The plausibility (or designability) of sampled proteins is clearly important because we want our designs to fold successfully in solution and can be evaluated using self-consistency metrics, which predict the structure of a designed sequence for a protein and assess the agreement between the predicted structure and sampled structure. The agreement is typically scored with either the RMSD metric or the TM-score metric calculated on the alpha-carbon atoms (referred to as scRMSD and scTM), and these metrics have been suggested to correlate with experimental success (13, 18, 21). We compute these scores on both the backbone and all-atom models using ESMFold as a structure prediction oracle and ProteinMPNN as a sequence design model (54, 57), noting a moderate effect of the number of attempts on the metric (*SI Appendix*, Fig. S2). We can further assess the chemical quality of model generations by measuring quantities such as bond lengths, bond angles, and dihedral angles, and assessing whether they align with real proteins. In this vein, we also compute the mean bond length RMSE metric, which measures the mean deviation of each bond length from an ideal value, and which we find correlates with the other angle-based statistics.

The diversity of model samples is an important quality not only because we want to avoid artifacts of generative modeling (e.g., mode collapse, where models optimize their training objectives by producing a limited range of high-quality samples), but also because we want to draw deeply on protein structural space to produce solutions for different design problems. Diversity has previously been measured by clustering samples and counting them (18, 21), but these metrics are biased in and dependent on the number of samples drawn N: mode-counting metrics will interpolate from 1 when N=1 to 0 as N approaches infinity. We compute a variant of the mean max-TM used in ref. 20 which is simply the mean over all pairwise TM scores between samples. This gives an unbiased estimate of the true mean pairwise TM, with only the variance decreasing as N approaches infinity. We also computed the secondary structure content of samples with DSSP, measuring whether samples cover a broad range of alpha and beta-type structures (58). Finally, to assess whether our model is able to generalize beyond the dataset (i.e., to evaluate novelty), we measured the TM-score of each sample against its nearest neighbor in the dataset. This nnTM metric indicates whether a sample is memorized or reproduced from the dataset, and describes the model’s ability to produce entirely novel proteins, relative to its training set.

**Fig. 2.**
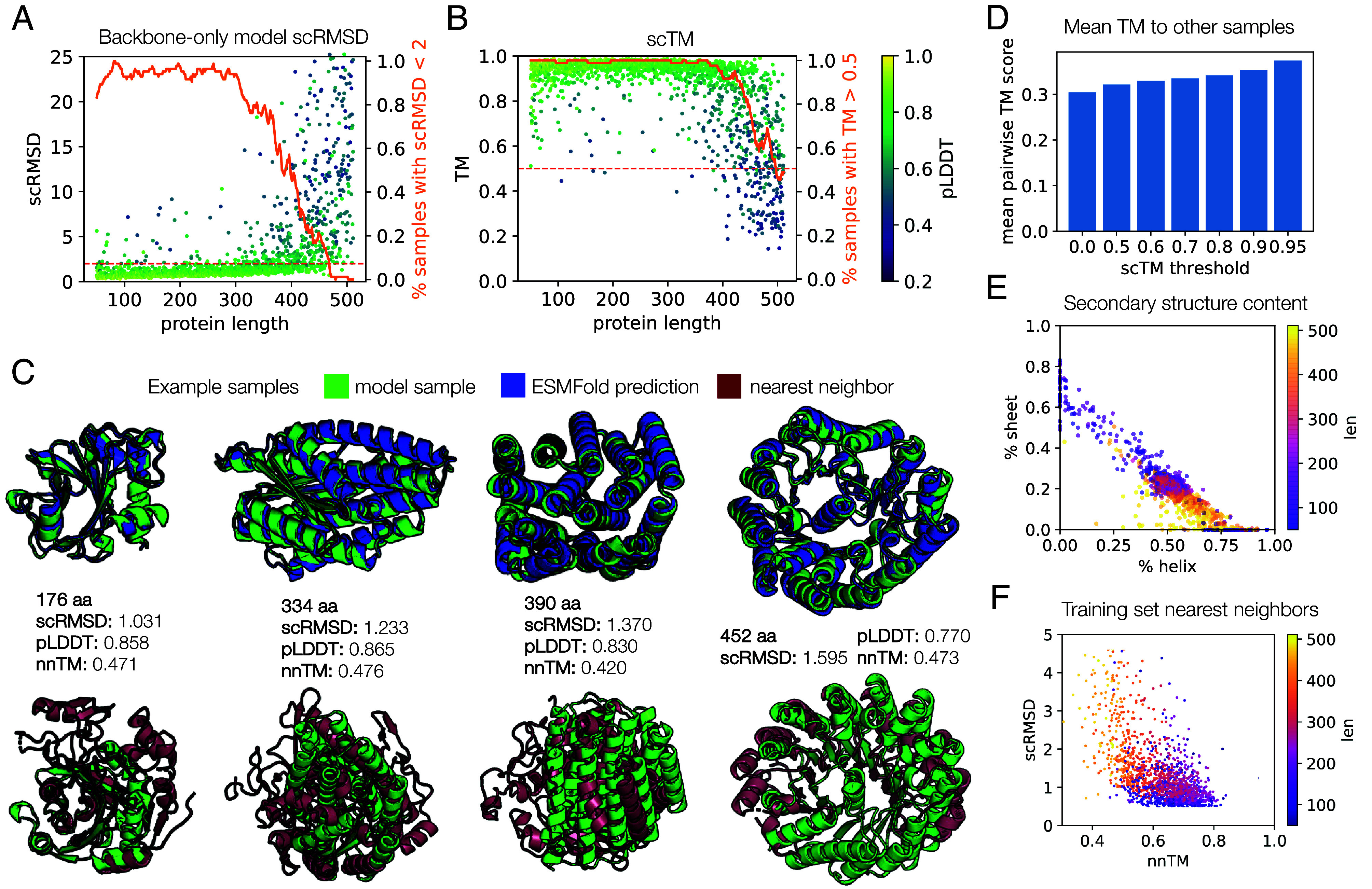
Evaluation of proteins sampled from the backbone-only model. (*A*) Self-consistency performance. We show the RMSD = 2 threshold (dashed line) and the proportion of samples passing this threshold, smoothed with a sliding window of size 21 (solid line). Eight backbones were sampled for each length from 50 to 512. For each backbone, the best of 8 ProteinMPNN-designed sequences is selected and ESMFold is used for all structure predictions. (*B*) The same samples and ESMFold predictions as in (*A*), but using the scTM metric. TM is computed using the same alignment as for RMSD. The dashed line indicates TM = 0.5; the solid line indicates proportion of samples with TM > 0.5, smoothed with sliding window of size 11. (*C*) Example high-quality, novel backbone model samples in green, shown aligned to the ESMFold prediction (blue) and the nearest neighbor in the dataset (red). The lengths, scRMSD, pLDDT, and nnTM metrics for each sample are also shown. (*D*) The mean over all pairwise TM scores is plotted for all samples (threshold = 0.0), and samples filtered to those with scTM greater than the indicated threshold. Lower values indicate more diversity. (*E*) Secondary structure content of samples, computed by DSSP. (*F*) Nearest neighbor distances for model samples with scRMSD < 5. The nnTM is the TM score against the dataset member with the highest TM score to the sample.

The backbone model, when combined with ProteinMPNN as the sequence design module, achieves strong performance on these properties. Our sampling-time tuning and optimization allow us to unconditionally generate proteins of length up to 500 with comparable designability to current methods, at a much lower computational cost (Fig. 2*A*–*C* and *SI Appendix*, Fig. S3 and Table S3). Our success rate under both the scRMSD and scTM metrics is ≥90% for most of this length range, dropping to ∼70% at the top end, while generating samples two orders of magnitude faster than other current methods: ∼0.15 s/100 residues compared to ∼15–60 s/100 residues for Chroma, FrameDiff, and RFdiffusion (*SI Appendix*, Fig. S3 and Table S3). Samples are also quite diverse, covering a broad range of alpha-beta content proportions (Fig. 2 *D* and *E*). With respect to novelty, given enough samples, the backbone model is able to generate proteins that belong to unique folds compared to those found in the training set (with TM < 0.5) (Fig. 2*F*). However, most nnTM values fall between 0.6 and 0.8, indicating that most samples share a common fold with a dataset member, but never just reproduce training examples. Comparing to other backbone generative models, we achieve performance comparable with the state of the art (RFdiffusion) on unconditional backbone generation, in particular achieving better designability with slightly worse diversity and novelty on proteins shorter than 200 residues, with two orders of magnitude reduction in sampling time (*SI Appendix*, Table S3).

Since the backbone-only model solves the probability flow ODE, it is also possible to frame it as a CNF to deterministically produce latent encodings of data and compute log-likelihoods (though note that we do not train with a maximum likelihood criterion). In particular, our model likelihoods are far faster to compute than self-consistency metrics, suggesting a more efficient way to filter for high-quality designs. We curated a set of samples from both the backbone-only and all-atom models produced during evaluation which had associated self-consistency metrics already computed and computed backbone-only likelihoods for these samples. We observed a correlation of likelihoods with scRMSD, scTM, and pLDDT and find that filtering samples offers a fast way to trim low-quality tails of the scRMSD/pLDDT distributions (*SI Appendix*, Figs. S4 and S5). In particular, likelihoods below a threshold (roughly 5 nats per atom) seem to be good at identifying lower quality samples. This aligns with computed likelihoods for the validation and test sets, where we rarely assign likelihoods of less than 5 nats per atom to natural protein structures, and observe similar values as for Chroma on the ELBO (*SI Appendix*, Fig. S6) (17). We did not observe a correlation between self-consistency and likelihood for natural proteins, perhaps because the differences in self-consistency for natural proteins (whose structures are known to be good) may reflect more on idiosyncrasies of the sequence design and structure prediction models than on the actual quality of the structures.

We assessed the all-atom model on the same metrics to evaluate its ability for unconditional protein generation. Sampling is fairly robust at lengths up to 250, with a success rate of ∼60% on proteins in this range when assessed by scRMSD (*SI Appendix*, Fig. S7 *A* and *B*). We are able to retain high sampling speeds, though the model is approximately ten-fold slower than the backbone-only model, due to the need to run miniMPNN and extra steps to allow more frequent visiting of different sidechain states (*SI Appendix*, Tables S2 and S4). Compared to the backbone model, the all-atom model appears less robust at all lengths, even when comparing only one ProteinMPNN sequence designed for each backbone-only sample (*SI Appendix*, Fig. S2*A*). This suggests that modeling protein backbones becomes more difficult for the all-atom model, perhaps because of the additional complexity of modeling the sidechain atoms. This effect can be observed when inspecting the absolute values of the train and validation losses, which are higher in general for the all-atom denoiser (*SI Appendix*, Fig. S1). Despite the fact that we do not explicitly provide sequence to the model, it is possible that sample quality and/or diversity may be affected by sequence leakage (*SI Appendix*, section D).

**Fig. 3.**
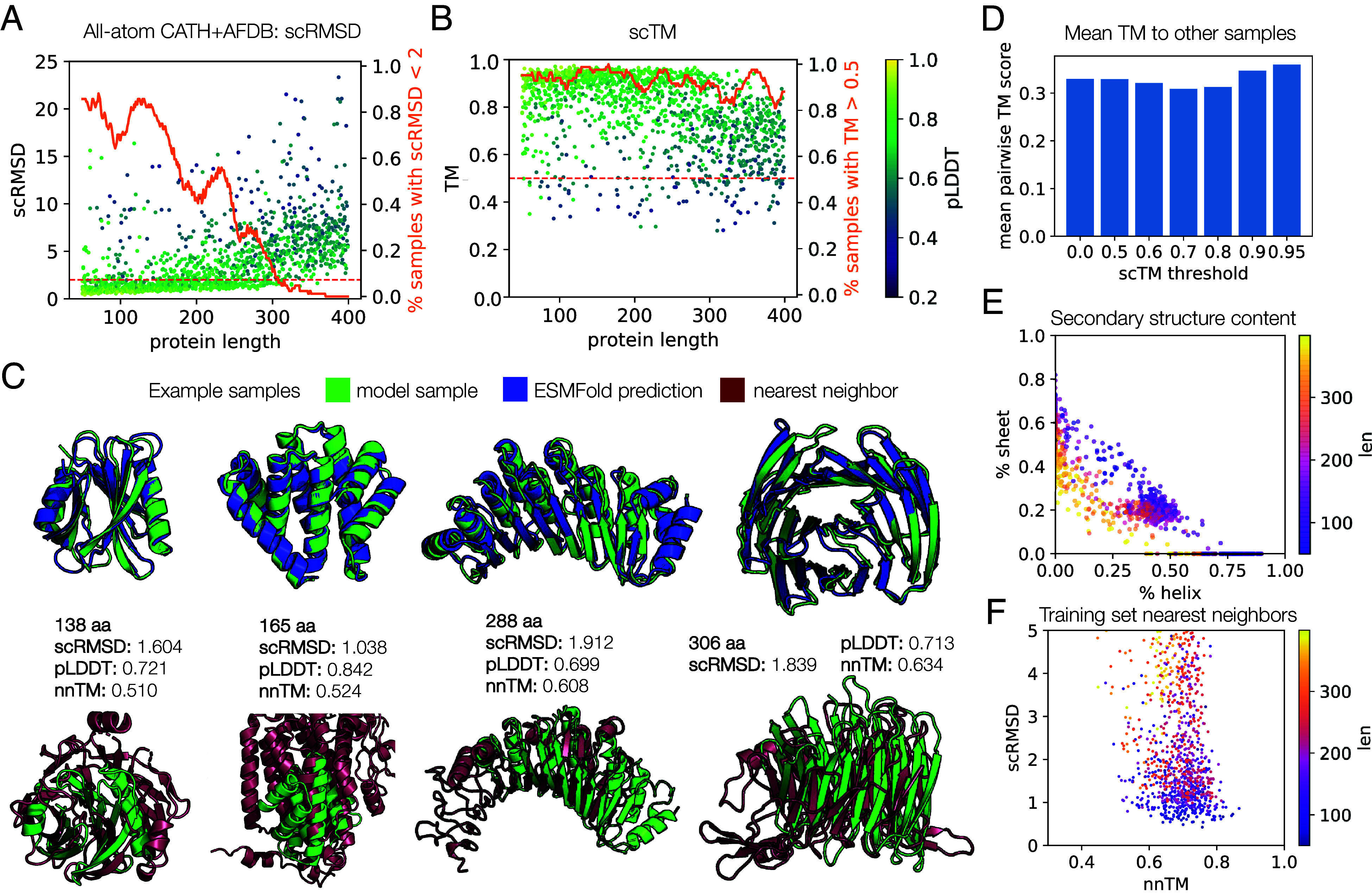
Evaluation of proteins sampled from the all-atom model trained on CATH + AFDB. (*A*) Self-consistency performance computed as in Fig. 2, but for the all-atom model. Eight proteins were sampled for each length from 50 to 400. Each protein’s sequence is used for ESMFold, i.e., only one sequence is predicted for each sample, rather than the eight sequences per sample that were predicted for the backbone-only model samples. The success proportion line is smoothed with a sliding window of 21. (*B*) The same samples and ESMFold predictions as in (*A*), but using the scTM metric. (*C*) Example high-quality, novel all-atom model samples. (*D*) The mean over all pairwise TM scores is plotted for all samples (threshold = 0.0), and samples filtered to those with scTM greater than the indicated threshold. Lower values indicate more diversity. (*E*) Secondary structure content of samples, computed by DSSP. (*F*) Nearest neighbor distances for model samples with scRMSD < 5. The nnTM is the TM score against the CATH training set member with the highest TM score to the sample.

The model is also able to generate proteins of all compositions (*SI Appendix*, Fig. S7 *C* and *E*). However, we notice a stronger relative preference for proteins with roughly 50% helix and 20% sheet content. This could be due to overfitting to the dataset at certain lengths where data are more scarce (*SI Appendix*, Fig. S2*B*), sequence leakage, or other factors, and is a direction for further investigation. To explore the effect of the diversity of training data on this phenomenon, we trained another model on both CATH and the AlphaFold Protein Structure Database (AFDB) (59) (*SI Appendix*, section C), and observed that we are able to retain a similar level of generation quality (Fig. 3 *A* and *B*). However, we observe improved diversity of generations relative to the CATH-only model (Fig. 3*C*–*E*), suggesting that data diversity is important for coverage of the protein structural distribution, and additional data may help improve model performance in this aspect (*SI Appendix*, section C and Table S4). When searching for training set nearest neighbors, we see the same overall pattern as observed for the backbone model, with few memorized samples and few completely novel samples (Fig. 3*F*).

**Fig. 4.**
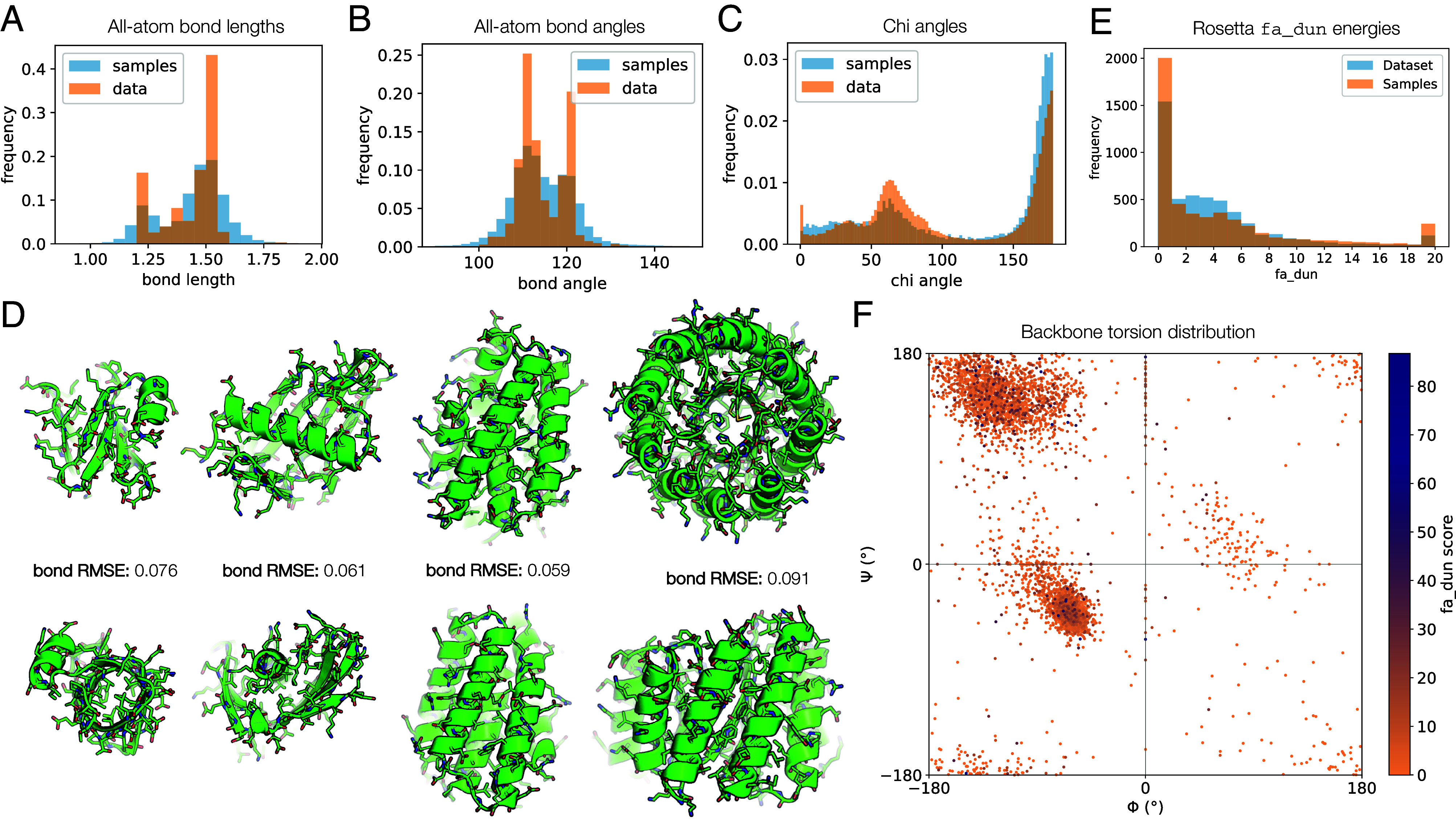
Analysis of generated all-atom structures, including sidechains. (*A*–*C*) Comparison of distributions of (*A*) bond lengths, (*B*) bond angles, and (*C*) chi angles for training data and model samples. Quantities for real data are computed from 100 random proteins from the training set; quantities for model samples are computed from 1 sample for each even-numbered protein length from 50 to 256. These results are computed on samples from an older checkpoint trained on CATH only, but the same chemical quality results hold for newer checkpoints including those trained on AFDB. (*D*) Detailed views of all-atom raw model output with sidechains built. The bond length RMSE is shown, which is computed by averaging the RMSE between each individual bond length and an idealized bond length in angstroms. For comparison, unrelaxed structures of natural proteins typically have an average bond length RMSE of 0.01 to 0.02. (*E*) Distribution of fa_dun energies for model samples and natural proteins. Statistics are computed from 5,000 residues chosen at random (without regard to individual proteins) each from the dataset and the set of model samples. The fa_dun energy is computed from the probability of a rotamer given the backbone torsions and a potential term for deviation from an ideal chi angle value. (*F*) Visualizing the model samples data from (*E*) on a Ramachandran plot. Each point is a pair of residue backbone torsions, colored by the fa_dun Rosetta energy.

The previously discussed metrics can be computed solely on a structure (i.e., backbone) and the corresponding sequence. Since our model generates the atoms of the sidechains independently and does not enforce idealized bond geometries, we further evaluated the chemical quality of the samples after two-stage sampling, which improves sidechain quality significantly (*SI Appendix*, Figs. S8 and S9). When compared to the ground truth training data, we find that samples from the model generally follow the same distribution with the same modes for bond lengths and bond angles, but with greater variance, as is often the case with free-atom generation methods (Fig. 4 *A* and *B*). We conduct these analyses without relaxing model samples or the dataset under an energy function such as Rosetta, since the noising data augmentation in diffusion destroys this information anyway (60–62) (*SI Appendix*, section C). When examining the chi angles, the model samples are able to capture the two main modes of the natural distribution (Fig. 4*C*). The model distribution is more smoothed at lower values, missing one of the smaller modes and showing greater density than natural proteins in some regions. When we visually examine the generated structures, they appear plausible, exhibiting convincing packing and sidechain rotamers, and in some cases reproduce specific sidechain interactions seen in natural proteins, such as salt bridges between charged surface residues, helix capping, and some hydrogen bonding interactions (Fig. 4*D*).

We also wanted to examine some of these properties statistically and explore whether the model learns to reproduce the backbone-dependent rotamer distributions observed in natural proteins and recorded in the Dunbrack rotamer libraries (63). For each residue in a set of model samples, we computed the backbone phi-psi torsion angles and the fa_dun Rosetta energy term (a score derived from the probabilities of the rotamers and harmonic potentials for the chi angles, given the phi-psi backbone torsions). Even without Rosetta energy minimization (relax), which is typically needed for optimal Rosetta energy values, most residues score within a tolerable range for the fa_dun energy, and closely follow the distribution of (unrelaxed) natural protein structures (Fig. 4*E*). Overall, the model samples obey proper chirality rules and exhibit backbone torsion distributions similar to native proteins (Fig. 4*F*). Outliers in fa_dun energy do not seem to correlate with any particular backbone torsion bin, suggesting that the model can generate sidechains for all forms of secondary structure well (Fig. 4*F*). We note that the fa_dun energy term can be noisy; in some cases, the score is very high, but this is also observed in natural proteins (Fig. 4*E*).

Our two-stage sampling procedure which includes a second stage of sidechain refinement procedure indicated that as a general model of all-atom protein structure, our model might be intrinsically capable of full-atom protein design tasks, such as fast flexible-backbone sidechain repacking. To investigate this, we measured the performance of our model on repacking tasks without any additional training or fine-tuning and compared against existing methods such as Rosetta, AttnPacker, and the Chroma packer on repacking de novo diffusion backbones, ESMFold predictions of these designs, and the CASP13 and CASP14 test sets (*SI Appendix*, Tables S5–S8) (17, 64, 65). The repacking-specific methods perform very strongly on these tasks. Despite the fact that our method is the only one which is not purposely developed for sidechain packing, we perform reasonably well overall, especially on design-relevant tasks such as repacking on diffusion-generated backbones and on the ESMFold predictions of these backbones (using ESMFold as a way of correcting any backbone pathologies). Notably, when backbones were allowed to adjust (e.g., “0.8-cond” samples), Protpardelle updated the backbone coordinates to improve the structures (*SI Appendix*, Tables S5 and S6 and Fig. S10). This indicates that the all-atom model is able to codesign structure and sequence, allowing the sidechains to influence the backbone and vice versa. Protpardelle repacking achieves a marginally higher clashing percentage than purpose-trained repacking methods (3.8% for 0.8-cond., relative to 1.3% AttnPack and 1.9% Chroma) due to inaccuracies in atom placement. A small subset of repacked sidechains showed distorted geometry and contributed to the ∼2% sidechain clash.

### Sidechain-Conditional Protein Design.

Our fundamental motivation was to develop a method for protein design that factors all-atom information into and throughout the entire design process, and to move toward design methods that allow for conditioning on arbitrary portions of protein structure, such as functional chemical groups, in a backbone- and rotamer-independent way. This allows combinatorially complex structural decisions, such as deducing the jointly optimal rotamers for each sidechain, to be incorporated directly into the model and solved in an end-to-end manner, rather than depending on external or hand-crafted solutions. Also, since backbone conditioning can lock in a particular backbone conformation or fold family without allowing the flexibility of sidechain conformations to allow searching over different fold spaces, sidechain-conditional generation might allow more diverse fold solutions to scaffolding problems. To this end, we explored whether the model has potential for designing new proteins in an all-atom manner. We trained a preliminary, crop-conditional model by providing it with randomly selected residues. These crops included contiguous spans of residues and discontiguous yet proximal residues; for some of these examples, the backbone was masked so that only the sidechain, or a small part of the sidechain, was provided to the model.

**Fig. 5.**
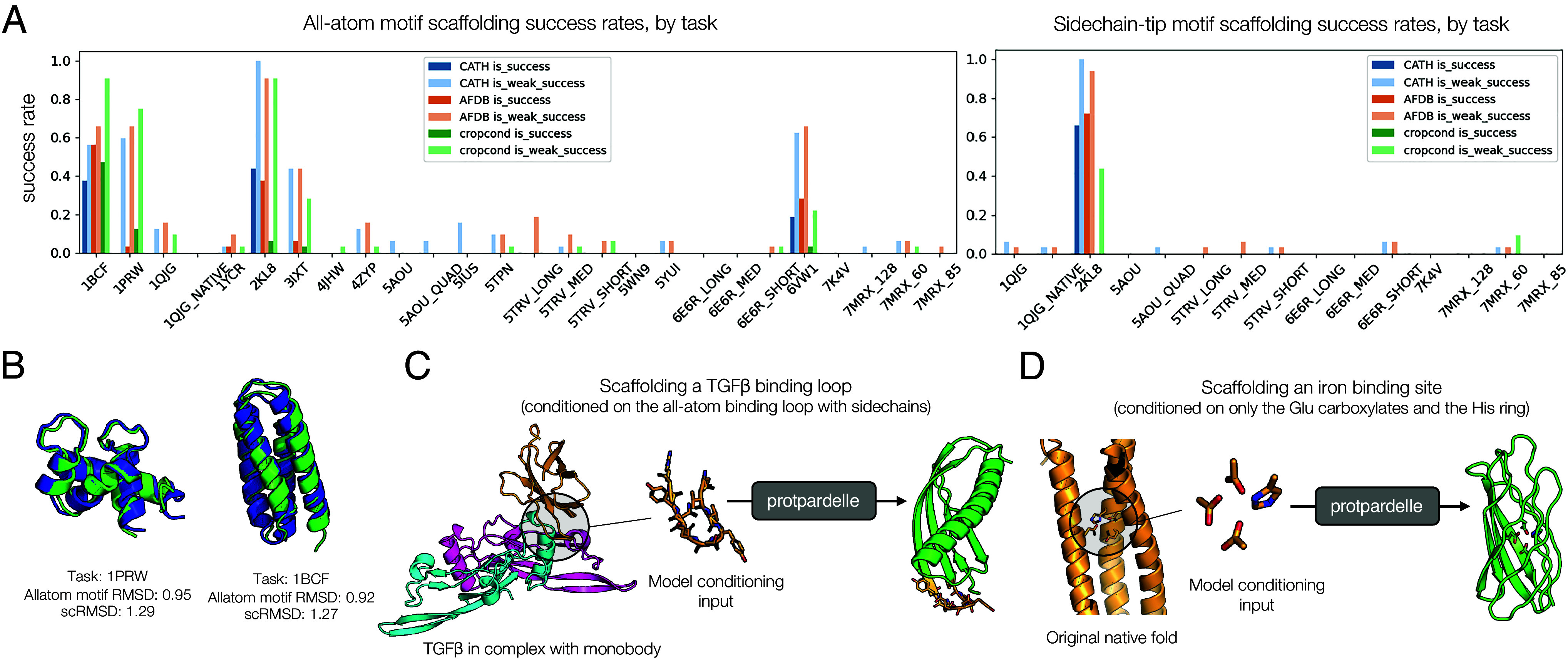
Towards all-atom protein design. (*A*) Performance of unconditional models (trained on CATH or CATH+AFDB) and a crop-conditional model trained on CATH on an augmented version of the RFdiffusion scaffolding benchmark. *Left*: scaffolding on all atoms of the motif. *Right*: scaffolding only the ends of fixed sidechains; the atoms after the final rotatable bond. For each model we sample with 40 steps of annealed MCMC and reconstruction guidance. We draw 32 samples for each task and report the success and weak-success rates. Successes are defined as all-atom motif RMSD < 2, backbone motif RMSD < 1, scRMSD < 2, and pLDDT > 70. Weak successes are defined as all-atom motif RMSD < 4, backbone motif RMSD < 3, scTM > 0.5. (*B*) Example successful all-atom scaffolding designs. (*C* and *D*) Potential applications of our model for new approaches to protein design. The conditioning portion is shown in gold on the model sample (green indicates the model-generated portion). These designs are generated with an initial crop-conditional model and reconstruction guidance. (*C*) An example design generated by scaffolding a TGF-β1 binding loop including its sidechains. The original binder design (in gold) is a de novo designed monobody and thus is guaranteed not to be in the training set. The pink and cyan chains are TGF-β1 (PDB: 4KV5). (*D*) An example design generated by scaffolding only the functional groups of iron-binding Glu and His residues. The model is given only the atoms after the last chi angle: (CG, CD, OE1, OE2) for the Glu residues and (CB, CG, CD2, CE1, ND1, NE2) for the His residues. The native fold shown is chain A of 1BCF.

We briefly explored inpainting and scaffolding motifs at a backbone-only level, and find that we are able to generate reasonable inpainting designs (*SI Appendix*, Fig. S11). To more deeply investigate the capacity of the all-atom model to perform motif scaffolding, we evaluated the performance of this model and of the unconditional models on the scaffolding benchmark used for RFjoint and RFdiffusion (11, 18). This benchmark contains a set of scaffolding tasks which are centered on placing motifs enriched in secondary structure in the context of a “foldable” protein scaffold. Simple replacement guidance to perform conditional sampling with either the unconditional or crop-conditional models proved difficult, except on easy tasks such as inpainting (2KL8), so we explored other strategies to improve conditional generation. We found that a combination of reconstruction guidance and annealed MCMC with a Metropolis correction provided the best results (39, 66, 67) (*SI Appendix*, section G and Fig. S12). Measuring both a strict and weak form of success rate for each task, we find that we are able to obtain at least some weak successes on most tasks when all-atom conditioning is enabled, and slightly less often when conditioning only on sidechain tip atoms of motifs (Fig. 5 *A* and *B*).

We also considered two specially targeted cases of all-atom conditional generation. To test our ability to design new protein–protein interactions, we explored scaffolding a protein-binding motif in an all-atom manner, providing as input the backbone, sidechains, and sequence of only the motif. For this, we selected a de novo monobody designed to bind TGF-β1, a cytokine that activates a receptor kinase with multiple downstream signaling targets (PDB: 4KV5). Since this protein is designed de novo by the Sculptor algorithm (19) and does not have a crystal structure in the PDB, it is guaranteed to not have been seen by the model during training. We extracted a loop and its sidechains from the binding motif of the monobody as conditioning input to the model and generated samples using Protpardelle. The model generates protein structures that plausibly scaffold this motif by eye and exhibit folds different from the original monobody, though these designs do not yet pass ESMFold-based self-consistency thresholds (Fig. 5*C*).

A method to generate protein structures in an all-atom manner also enables alternative ways to generate proteins conditioned on functional motifs. Most current machine learning-based methods rely on models to first infer backbone conformations that seem mutually probable with the binding targets, and then to design the sequence and model the sidechains. We wanted to explore whether it is possible to generate complete proteins conditioned directly on the chemical groups which mediate the interaction. A metal-binding interaction is primarily dependent on the ligand interactions formed by the polarizable groups on the surrounding sidechains. We extracted only these polarizable groups for a single metal from a natural diiron-binding protein, cytochrome b1 (PDB: 1BCF); these groups included the carboxylates on three glutamate residues and the imidazole ring from the histidine residue. With only the atoms of these specific groups as conditioning input to the model, the model was able to design a protein scaffold that hosts these groups, together with the remaining sidechain atoms and rotamers needed (Fig. 5*D*). Some designed folds also differ from the original native fold. As with the monobody binder, we found these structures to be mostly plausible by inspection, but not yet ESMFold self-consistent. This suggests that our model could be an approach to designing functional proteins in a backbone- and rotamer-free manner.

In these cases, we are able to obtain solutions resembling protein structures by eye, demonstrating the ability of the method to explore new scaffolding solutions. For example, solutions to the 1BCF task are almost always four-helix bundles when scaffolded with backbone-only models; however, in our setup, we are able to generate a beta-barrel-like structure to scaffold these motifs when conditioned only on the sidechain tips. This indicates that our method could be a promising approach for diversifying protein design solutions. However, fidelity to conditioning, sampling success rate, and overall quality of these conditional generations is lower than for backbone-only scaffolding methods and remains to be improved. Many protein design tasks can be posed as some form of inpainting or outpainting; training crop-conditional models has been a successful strategy for inpainting and scaffolding continuous and discontinuous functional sites elsewhere (11, 14, 18, 24). We expect this to hold for Protpardelle, as well as the many other tactics for applying conditioning and improving cycle consistency in score-based generative models (68, 69).

## Conclusion

Our results describe a number of contributions that we hope will advance the field of protein design. We have applied a diffusion process and network architecture, both originally developed in computer vision, to protein structures in a way that allows for high-quality sampling of proteins. We describe a way to alter this process during sampling with a sidechain superposition so that a model trained only on standard protein structures is able to denoise sidechains for arbitrary sequences that any sequence design model might choose. This enables all-atom protein generation in a way where the model is able to reason about the sidechains jointly with the backbone, since both are noised and denoised together.

We have also conducted preliminary exploration of potential future applications for our model. These include fast flexible-backbone sidechain repacking and generating new proteins and designing proteins conditioned on explicit sidechain interactions. Other applications may yet be interesting to explore. Possibly the free-atom representation we use facilitates learning more physically realistic interactions to be modeled than popular abstractions such as residue frames, and together with the deterministic ODE formulation may enable more investigations of the relationships between model likelihoods or latent embeddings and the energy landscape of protein structures. We believe these to be interesting directions for future methods development.

We stress that while our model is capable of codesigning sequence and structure, it remains a structure-primary generative model that produces estimates of the sequence during its sampling trajectory. It does not define any noising process on the sequence; nor is it a joint model in the sense that we are able to marginalize and condition in some way to produce solutions to the subtasks of structure and sequence generation and forward and inverse folding. However, structure-primary approaches have shown ever-increasing capabilities to generate proteins with novel functions (18). We hope that as sequence codesign and all-atom modeling become integrated, as we have shown here ( 70), effective new ways to solve difficult protein design goals can be found.

## Supplementary Material

Appendix 01 (PDF)

## Data Availability

Code and model parameters’ data have been deposited in GitHub (https://github.com/ProteinDesignLab/protpardelle) (70).

## References

[1] P. S. Huang, S. E. Boyken, D. Baker, The coming of age of de novo protein design. Nature **537**, 320–327 (2016). 27629638 10.1038/nature19946

[2] I. V. Korendovych, W. F. DeGrado, De novo protein design, a retrospective. Q. Rev. Biophys. **53**, e3 (2020). 32041676 10.1017/S0033583519000131 PMC7243446

[3] P. S. Huang , Rosettaremodel: A generalized framework for flexible backbone protein design. PLoS ONE **6**, e24109 (2011). 21909381 10.1371/journal.pone.0024109 PMC3166072

[4] N. Anand, P. Huang, “Generative modeling for protein structures” in *Advances in Neural Information Processing Systems*, S. Bengio , Eds. (Curran Associates, Inc., 2018), vol. 31, (2018).

[5] N. Anand, R. Eguchi, P. S. Huang, Fully differentiable full-atom protein backbone generation. ICLR 2019 Workshop DeepGenStruct (2019). https://openreview.net/forum?id=SJxnVL8YOV. Accessed 6 June 2024.

[6] R. R. Eguchi, C. A. Choe, P. S. Huang, Ig-VAE: Generative modeling of protein structure by direct 3D coordinate generation. PLOS Comput. Biol. **18**, e1010271 (2022). 35759518 10.1371/journal.pcbi.1010271 PMC9269947

[7] Z. Lin, T. Sercu, Y. LeCun, A. Rives, Deep generative models create new and diverse protein structures. NeurIPS MLSB workshop (2021). https://www.mlsb.io/papers_2021/MLSB2021_Deep_generative_models_create.pdf. Accessed 6 June 2024.

[8] I. Anishchenko , De novo protein design by deep network hallucination. Nature **600**, 547–552 (2021). 34853475 10.1038/s41586-021-04184-w PMC9293396

[9] D. Tischer *et al*., Design of proteins presenting discontinuous functional sites using deep learning. bioRxiv [Preprint] (2020). 10.1101/2020.11.29.402743 (Accessed 6 June 2024).

[10] B. Huang , A backbone-centred energy function of neural networks for protein design. Nature **602**, 523–528 (2022). 35140398 10.1038/s41586-021-04383-5

[11] J. Wang , Scaffolding protein functional sites using deep learning. Science **377**, 387–394 (2022). 35862514 10.1126/science.abn2100 PMC9621694

[12] N. Anand, T. Achim, Protein structure and sequence generation with equivariant denoising diffusion probabilistic models. arXiv [Preprint] (2022). 10.48550/arXiv.2205.15019 (Accessed 6 June 2024).

[13] B. L. Trippe ., Diffusion probabilistic modeling of protein backbones in 3D for the motifscaffolding Problem. arXiv [Preprint] (2022). https://arxiv.org/abs/2206.04119 (Accessed 6 June 2024).

[14] J. S. Lee, J. Kim, P. M. Kim, Score-based generative modeling for de novo protein design. bioRxiv [Preprint] (2023). 10.1101/2022.07.13.499967 (Accessed 6 June 2024). 38177840

[15] C. Shi, C. Wang, J. Lu, B. Zhong, J. Tang, Protein sequence and structure co-design with equivariant translation. ICLR (2023). https://openreview.net/forum?id=pRCMXcfdihq. Accessed 6 June 2024.

[16] K. E. Wu , Protein structure generation via folding diffusion. Nat. Commun. **15**, 1059 (2022). 10.1038/s41467-024-45051-2 PMC10844308 38316764

[17] J. Ingraham , Illuminating protein space with a programmable generative model. Nature **623**, 1070–1078 (2023). 37968394 10.1038/s41586-023-06728-8 PMC10686827

[18] J. L. Watson *et al*., Broadly applicable and accurate protein design by integrating structure prediction networks and diffusion generative models. bioRxiv [Preprint] (2022). 10.1101/2022.12.09.519842 (Accessed 6 June 2024).

[19] R. R. Eguchi *et al*., Deep generative design of epitope-specific binding proteins by latent conformation optimization. bioRxiv [Preprint] (2022). 10.1101/2022.12.22.521698 (Accessed 6 June 2024).

[20] Y. Lin, M. AlQuraishi, Generating novel, designable, and diverse protein structures by equivariantly diffusing oriented residue clouds. arXiv [Preprint] (2023). 10.48550/arXiv.2301.12485 (Accessed 6 June 2024).

[21] J. Yim , “SE(3) diffusion model with application to protein backbone generation” in *Proceedings of the 40th International Conference on Machine Learning, PMLR* (2023), vol. 202, pp. 40001–40039.

[22] B. Hie *et al*., A high-level programming language for generative protein design. bioRxiv [Preprint] (2022). 10.1101/2022.12.21.521526 (Accessed 6 June 2024).

[23] R. Verkuil *et al*., Language models generalize beyond natural proteins. bioRxiv [Preprint] (2022). 10.1101/2022.12.21.521521 (Accessed 6 June 2024).

[24] S. L. Lisanza *et al*., Joint generation of protein sequence and structure with rosettafold sequence space diffusion. bioRxiv [Preprint] (2023). 10.1101/2023.05.08.539766 (Accessed 6 June 2024).

[25] W. Jin, J. Wohlwend, R. Barzilay, T. Jaakkola, Iterative refinement graph neural network for ntibody sequence-structure co-design. arXiv [Preprint] (2022). https://arxiv.org/abs/2110.04624 (Accessed 6 June 2024).

[26] X. Kong, W. Huang, Y. Liu, Conditional antibody design as 3d equivariant graph translation. arXiv [Preprint] (2023). https://arxiv.org/abs/2208.06073 (Accessed 6 June 2024).

[27] S. Luo *et al*., Antigen-specific antibody design and optimization with diffusion-based generative models for protein structures. bioRxiv [Preprint] (2022). 10.1101/2022.07.10.499510 (Accessed 6 June 2024).

[28] K. Gao , Incorporating pre-training paradigm for antibody sequence-structure co-design. bioRxiv [Preprint] (2022). https://www.biorxiv.org/content/10.1101/2022.11.14.516404v2.full.pdf (Accessed 6 June 2024).

[29] X. Kong, W. Huang, Y. Liu, “End-to-end full-atom antibody design” in *Proceedings of the 40th International Conference on Machine Learning, PMLR* (2023), vol. 202, pp. 17409–17429.

[30] J. Sohl-Dickstein, E. A. Weiss, N. Maheswaranathan, S. Ganguli, “Deep unsupervised learning using nonequilibrium thermodynamics” in *the 32nd International Conference on Machine Learning, PMLR* (2015), vol. 37, pp. 2256–2265.

[31] J. Ho, A. Jain, P. Abbeel, Denoising diffusion probabilistic models. arXiv [Preprint] (2020). https://arxiv.org/abs/2006.11239 (Accessed 6 June 2024).

[32] Y. Song, S. Ermon, Generative modeling by estimating gradients of the data distribution. Adv. Neural Inf. Process. Syst. **32** (2019).

[33] Y. Song , Score-based generative modeling through stochastic differential equations. arXiv [Preprint] (2021). https://arxiv.org/abs/2011.13456 (Accessed 6 June 2024).

[34] P. Dhariwal, A. Nichol, Diffusion models beat GANs on image synthesis. arXiv [Preprint] (2021). https://arxiv.org/abs/2105.05233 (Accessed 6 June 2024).

[35] R. Aditya , “Zero-shot text-to-image generation” in *Proceedings of the 38th International Conference on Machine Learning, PMLR* (2021), vol. 139, pp. 8821–8831.

[36] J. Ho , Imagen video: High definition video generation with diffusion models. arXiv [Preprint] (2022). https://arxiv.org/abs/2210.02303 (Accessed 6 June 2024).

[37] J. Ho, T. Salimans, Classifier-free diffusion guidance. arXiv [Preprint] (2022). https://arxiv.org/abs/2207.12598 (Accessed 6 June 2024).

[38] A. Nichol , “Glide: Towards photorealistic image generation and editing with text-guided diffusion models” in *Proceedings of the 39th International Conference on Machine Learning, PMLR* (2022), vol. 162, pp. 16784–16804.

[39] J. Ho , Video diffusion models. arXiv [Preprint] (2022). *https://arxiv.org/abs/2204.03458* (Accessed 6 June 2024).

[40] A. Hyvärinen, Estimation of non-normalized statistical models by score matching. J. Mach. Learn. Res. **6**, 695–709 (2005).

[41] P. Vincent, A connection between score matching and denoising autoencoders. Neural Comput. **23**, 1661–1674 (2011). 21492012 10.1162/NECO_a_00142

[42] L. Dinh, J. Sohl-Dickstein, S. Bengio, Density estimation using Real NVP. arXiv [Preprint] (2017). https://arxiv.org/abs/1605.08803 (Accessed 6 June 2024).

[43] D. P. Kingma, P. Dhariwal, Glow: Generative flow with invertible 1x1 convolutions. arXiv [Preprint] (2018). https://arxiv.org/abs/1807.03039 (Accessed 6 June 2024).

[44] T. Karras, M. Aittala, T. Aila, S. Laine, Elucidating the design space of diffusion-based generative models. arXiv [Preprint] (2022). https://arxiv.org/abs/2206.00364 (Accessed 6 June 2024).

[45] J. Song, C. Meng, S. Ermon, Denoising diffusion implicit models. arXiv [Preprint] (2021). https://arxiv.org/abs/2010.02502 (Accessed 6 June 2024).

[46] R. J. McCann, A convexity principle for interacting gases. Adv. Math. **128**, 153–179 (1997).

[47] M. S. Albergo, N. M. Boffi, E. Vanden-Eijnden, Stochastic interpolants: A unifying framework for flows and diffusions. arXiv [Preprint] (2023). https://arxiv.org/abs/2303.08797 (Accessed 6 June 2024).

[48] X. Liu, C. Gong, Q. Liu, Flow straight and fast: Learning to generate and transfer data with rectified flow. arXiv [Preprint] (2022). https://arxiv.org/abs/2209.03003 (Accessed 6 June 2024).

[49] Y. Lipman, R. T. Q. Chen, H. Ben-Hamu, M. Nickel, M. Le, Flow matching for generative modeling. arXiv [Preprint] (2023). https://arxiv.org/abs/2210.02747 (Accessed 6 June 2024).

[50] M. Xu , “Geodiff: A geometric diffusion model for molecular conformation generation” in *International Conference on Learning Representations* (2022).

[51] C. B. Anfinsen, Principles that govern the folding of protein chains. Science **181**, 223–230 (1973). 4124164 10.1126/science.181.4096.223

[52] A. Campbell , Trans-dimensional generative modeling via jump diffusion models. arXiv [Preprint] (2023). https://arxiv.org/abs/2305.16261 (Accessed 6 June 2024).

[53] E. Hoogeboom, J. Heek, T. Salimans, “Simple diffusion: End-to-end diffusion for high resolution images” in *Proceedings of the 40th International Conference on Machine Learning, PMLR* (2023), vol. 202, pp. 13213–13232.

[54] J. Dauparas , Robust deep learning–based protein sequence design using proteinMPNN. Science **378**, 49–56 (2022). 36108050 10.1126/science.add2187 PMC9997061

[55] E. Perez, F. Strub, H. de Vries, V. Dumoulin, A. Courville, “Film: Visual reasoning with a general conditioning layer”. arXiv [Preprint] (2018). https://arxiv.org/abs/1709.07871 (Accessed 6 June 2024).

[56] T. Chen, R. Zhang, G. Hinton, Analog bits: Generating discrete data using diffusion models with self-conditioning. arXiv [Preprint] (2023). https://arxiv.org/abs/2208.04202 (Accessed 6 June 2024).

[57] Z. Lin , Evolutionary-scale prediction of atomic-level protein structure with a language model. Science **379**, 1123–1130 (2023). 36927031 10.1126/science.ade2574

[58] W. Kabsch, C. Sander, Dictionary of protein secondary structure: Pattern recognition of hydrogen-bonded and geometrical features. Biopolymers **22**, 2577–2637 (1983). 6667333 10.1002/bip.360221211

[59] M. Varadi , AlphaFold Protein Structure Database: Massively expanding the structural coverage of protein-sequence space with high-accuracy models. Nucleic Acids Res. **50**, D439–D444 (2021). 10.1093/nar/gkab1061 PMC8728224 34791371

[60] R. F. Alford , The rosetta all-atom energy function for macromolecular modeling and design. J. Chem. Theory Comput. **13**, 3031–3048 (2017). 28430426 10.1021/acs.jctc.7b00125 PMC5717763

[61] P. Conway, M. D. Tyka, F. DiMaio, D. E. Konerding, D. Baker, Relaxation of backbone bond geometry improves protein energy landscape modeling. Protein Sci. **23**, 47–55 (2013). 10.1002/pro.2389 PMC3892298 24265211

[62] L. G. Nivón, R. Moretti, D. Baker, A pareto-optimal refinement method for protein design scaffolds. PLoS ONE **8**, 1–5 (2013). 10.1371/journal.pone.0059004 PMC3614904 23565140

[63] M. V. Shapovalov, R. L. Dunbrack, A smoothed backbone-dependent rotamer library for proteins derived from adaptive kernel density estimates and regressions. Structure **19** (6), 844–858 (2011). 21645855 10.1016/j.str.2011.03.019 PMC3118414

[64] A. Leaver-Fay , Rosetta3: An object-oriented software suite for the simulation and design of macromolecules. Methods Enzymol. **487**, 545–574 (2011). 21187238 10.1016/B978-0-12-381270-4.00019-6 PMC4083816

[65] M. McPartlon, J. Xu, An end-to-end deep learning method for protein side-chain packing and inverse folding. Proc. Natl. Acad. Sci. U.S.A. **120**, e2216438120 (2023). 37253017 10.1073/pnas.2216438120 PMC10266014

[66] J. Besag, Comments on “Representations of knowledge in complex systems” by U. Grenander and M. I. Miller. *J. R. Stat.* **56**, 549–603 (1994).

[67] Y. Du , “Reduce, reuse, recycle: Compositional generation with energy-based diffusion models and MCM” in *Proceedings of the 40th International Conference on Machine Learning, PMLR* (2023), vol. 202, pp. 8489–8510.

[68] A. Odena, C. Olah, J. Shlens, Conditional image synthesis with auxiliary classifier GANs. arXiv [Preprint] (2017). https://arxiv.org/abs/1610.09585 (Accessed 6 June 2024).

[69] J. Y. Zhu, T. Park, P. Isola, A. A. Efros, “Unpaired Image-to-Image Translation Using Cycle-Consistent Adversarial Networks” in *IEEE International Conference on Computer Vision (ICCV)* (2017), pp. 2223–2232.

[70] A. Chu , Protpardelle. Github. https://github.com/ProteinDesignLab/protpardelle. Deposited 7 September 2023.

