# Supplementary material for: An all-atom protein generative model
Source: Proc Natl Acad Sci U S A. 2024 Jun 25;121(27):e2311500121. doi: 10.1073/pnas.2311500121 (PMC11228509; doi:10.1073/pnas.2311500121)
Supplement: Supplementary file 1 — Appendix 01 (PDF) [file pnas.2311500121.sapp.pdf]

Supporting Information for

An all-atom protein generative model

Alexander E. Chu, Jinho Kim, Lucy Cheng, Gina El Nesr, Minkai Xu, Richard W. Shuai, and Po-Ssu Huang

Po-Ssu Huang
E-mail: possu@stanford.edu

This PDF file includes:

Supporting text
Figs. S1 to S15
Tables S1 to S9
SI References

Supporting Information Text

A. EM formulation. We can uncover another perspective on the superposition-based generation scheme by formulating it as pseudo-Expectation Maximization. For the all-atom modeling task in this paper, the data we want to generate is necessarily composed of both structure and residue information. (Here we differentiate *residue* information from *sequence* information; we only need the per-residue identities to identify sidechains, which might be predicted from structure and nearby amino acid identities, but in general we do not need the full joint distribution over sequences; see also supplement D for further discussion of the sequence leakage hypothesis.) During the training process for denoising, we fix the residue types and only learn the denoising dynamics over coordinates; that is we learn the transition kernels $p_\theta(x_{t-1}|x_t, r)$ conditioned on the residue information r. During the training process for sequence prediction, we fix the coordinates and learn the token distribution for each residue $q(r|x_t)$. Then for all-atom generation, we want to generate realistic protein structures with specified residue types and their atomic positions, given the learned transitions $p_\theta(x_{t-1}|x_t, r)$ conditioned on residue type information from $q(r|x_t)$. Taking the denoising network as $p_\theta(x_{t-1}|x_t, r)$ we can define the E and M steps as follows:

- **E-Step**: Set $p(x_{t-1}|x_t) = \mathbb{E}_{q(r|x_t)} p_\theta(x_{t-1}|x_t, r)$, where $q(r|x_t)$ denotes the operation of using miniMPNN to predict the residue types given the backbone of structure x_t at timestep t and then picking the corresponding atoms from the superpositions via collapse.

- **M-Step**: Sample $x_{t-1} \sim p(x_{t-1}|x_t)$, where x_t refers to the collapsed structure. The atoms might correspond to various noise levels, so we use varying step sizes to compute the structure update.

In this context we can see that the stepwise partial optimization of $p(x)$ (i.e. integrating the ODE for only a subset of superposition atoms at each step) is a form of coordinate descent on the structure superposition. These additional perspectives may guide future efforts for improving model performance and capabilities.

B. Network architectures. The structure network has a U-ViT architecture (1) with a hidden dimension of 256 (512 for the AFDB model) and is composed of 6 (8 for the AFDB model) residual noise-conditional transformer layers. The self-attention layers contain 8 attention heads of dimension 32, and the feedforward layers are 3-layer MLPs with an intermediate hidden dimension of 1024. Typically the U-ViT architecture is trained with convolutional down- and up-sampling layers, though we remove them; we find that adding these layers improves alpha helix generation but slightly diminishes the quality of beta sheets. We use a patch size of 1 x n_{atoms} so that each residue is its own patch. We apply preconditioning from Karras et al. which stabilizes network training (2). The noisy coordinate inputs to the model are given by $x + \epsilon$, where x is the N x 37 x 3 array of all atom coordinates (with zeros for non-existent atoms for a given residue identity), and ϵ is sampled from $\mathcal{N}(0, \sigma_t^2)$ independently for all N x 37 x 3 array elements, including atoms missing from a given residue. This obscures the sequence identities at all but low noise levels, at which point the model is only refining local chemical details. We also provide the 37 x 3 self-conditioning atom coordinates, the noise level, and the sequence mask (but not the atom mask). We use absolute position encodings but also experimented with relative and rotary embeddings and find them to work well. We found training with mixed precision and weight decay to be harmful to performance. For the crop-conditional model, we provide additional 37 x 3 crop-conditioning coordinates and center all noisy and clean coordinates together on the center of mass of the conditioning motif (or of the noisy protein, if no motif is provided). We explored providing the miniMPNN sequence prediction as input, but this had a minimal effect. Both the backbone-only model and the all-atom model are constructed with the same general configuration described here, with exception of the embedding layer which has a larger dimension in the all-atom model to account for the additional input atoms. We did not deeply explore the effect of tuning the network size hyperparameters.

To inject noise conditioning information, we apply a noise-dependent affine transformation (scale and shift) to network activations (3, 4). These are applied to the inputs of attention layers after normalizing, and to the intermediate representations of MLPs in feedforward layers, and in miniMPNN.

The miniMPNN model uses the same overall configuration as ProteinMPNN. We made two modifications: we removed the autogressive mask and added noise conditioning to the MLP blocks, and then trained it from scratch. We skewed the noise applied to the coordinates heavily towards 0 as we found that the model does not train effectively if it is frequently shown very noisy structures. While we currently use ProteinMPNN for the final step, we believe a mini-MPNN-only approach to be feasible as well; strong sequence design results have been obtained with non-autoregressive (albeit not noise-conditional) ProteinMPNN elsewhere (5).

C. Training details. We trained on the CATH S40 dataset which extracts domains from the PDB and removes redundant domains with $\geq 40\%$ sequence identity (6). We split the dataset into train, validation, and test sets using the splits in (7) and used the validation set to compute denoising loss metrics during training and evaluate the miniMPNN model. The backbone and all-atom models are trained on contiguous crops of length up to 512, uniformly sampled over the protein length for proteins longer than 512 residues. We do not relax the dataset under an energy function since the data augmentation associated with training diffusion models largely erases this information, although this can be explored in future work.

The model is trained with the Adam optimizer (8) with learning rate 1e-4 with a batch size of 32. We used linear warmup for the learning rate for 1000 steps followed by cosine decay. On a single NVIDIA A40 GPU, this takes approximate 4 days for 2.5M iterations for the backbone model, and 6 days for 2.5M iterations for the all-atom model. We note that this relatively low computational commitment indicates it may yet be possible to obtain significant performance improvements by scaling the model and dataset.

We also used a log-normal distribution $p_{train}(\sigma)$ over the noise levels which we find important for successful training (2). Noise levels applied to the data during training are sampled from a log-normal distribution over the noise levels, with $\mu = -1$ and $\sigma = 1.5$. In detail, we draw a sample from $\mathcal{N}(-1, 1.5^2)$, exponentiate it, and scale it by the standard deviation of the data to determine the scale of the Gaussian noise to add. (For the backbone model, we use $\mathcal{N}(-1.2, 1.2^2)$.) A unique noise level is sampled for each minibatch element. Data augmentation is applied by centering the protein structure at the mean alpha-carbon coordinates, sampling and applying a rotation matrix uniformly at random, and sampling and applying a random translation (3-vector) from the standard normal distribution.

Additionally denoising the sidechains might have a similar effect as diffusion modeling with high-resolution images; since there more atoms, more noise might be needed to destroy the same amount of information (9). We did not explore customized noise schedules, such as distinct or correlated noise schedules for backbone and sidechains, beyond a simple scaling of the noise scale for the sidechains that did not appear to be beneficial. Defining this relationship more clearly might enable tuning the trade-off between backbone and sidechain quality and the degree of interaction between the two during sampling.

For the model trained on a dataset augmented with structures from the AlphaFold Protein Structure Database (AFDB), we augmented our dataset by constructing a synthetic dataset from AlphaFold2 structure predictions downloaded from v4 of the AFDB (10). Foldseek has previously been applied to cluster over 214M structures from the AFDB into 15M structural clusters, 13M of which are singleton clusters (11, 12). We noticed that many singleton clusters exhibited less globular and more loopy structural features in a way that did not correlate with pLDDT or radius-of-gyration-based metrics (RG) and chose to eliminate them, hypothesizing that singleton clusters, by virtue of their dissimilarity to all other structures, are less likely to mirror the overall structural distribution we would like to model. To additionally filter for high quality structures, we took the representative structure of each non-singleton cluster and filtered for structures with an average pLDDT > 80. We also removed structures shorter than 50 residues or longer than 512 residues. Finally, noticing that many of the remaining structures were extended helices, following Verkuil et al., we filtered by each structure's RG relative to the idealized radius of a protein (13). To calculate the relative RG, we divided each structure's RG by $2.24 \cdot (N_{residues})^{0.392}$ as determined in (14). We filtered for structures whose relative RG was below 1.4, yielding a synthetic dataset with a total of approximately 584K AFDB structures (Supp. Fig. S13). During training, we sampled each batch such that 25% of the examples of each batch were randomly chosen from the CATH training set (approx. 18K structures) and 75% of examples were randomly chosen from our synthetic dataset. Since a larger dataset might require larger model capacity, we also increased the number of layers from 6 to 8 and the hidden dimension from 256 to 512. No tuning or optimization of any hyperparameters mentioned here was done.

D. Sequence leakage. In the main text we described a hypothesis which we call *sequence leakage*. The main concept is that it is possible for the denoising network to infer sequence from all-atom structure even when we don't explicitly provide the amino acid types. This is because the non-existent sidechain atoms for each residue in the N x 37 x 3 array (which we will call 'ghost atoms') are zeroed out by default, so the sidechain identities can be easily inferred (and thus the sequence). If the model is able to infer the sequence easily during training, this provides strong signal at high/moderate noise when denoising is difficult, and it is likely the model will try to use this signal to partially memorize structures given the sequence and further minimize the denoising loss. This is problematic because we don't have access to the ground truth sequence at sampling time, and cannot obtain good predictions of it until near the end ($t < 0.3$ or so) and the structure noise level is fairly low (so that the structure can be reliably denoised and MiniMPNN can predict a good sequence). However, by this point in the sampling procedure, the structure is also largely determined, so it cannot be remedied with the use of good sequence predictions. Thus a model *trained* to be dependent on "good" sequences at high/moderate noise will struggle with out-of-distribution "poor" sequences at high/moderate noise *during sampling* since we don't have "good" sequences at those timesteps. Early in model development with a model trained on zeroed-out ghost atoms, we noticed that generating an all-atom structure conditioned on a ground truth sequence (essentially, structure prediction) typically was able to reproduce secondary structure of the ground truth structure, but failed to produce the correct tertiary structure. This suggested that the model did infer some structural information from sequence, but lacked the correct inductive biases to map sequence to the appropriate structure, making it meaningless to condition on a sequence, even a ground truth sequence. Relatedly, generating all-atom structure conditioned on a sequence of all glycines (essentially, backbone generation) typically failed at generating realistic 'backbone-only' structures (good chemical quality, but poor secondary/tertiary organization). This also suggested that the model did depend on the sequence to some extent and was unable to strictly denoise "atoms", i.e. simply place atoms in the correct positions in space relative to each other without using the sequence.

Based on this hypothesis, we believed the best way to address sequence leakage was to render the denoising network agnostic to sequence as much as possible. We were able to improve performance by obscuring the sidechain identities by adding noise to rather than zeroing out the ghost atoms, reducing the model's ability to infer and become dependent on the sequence at high/moderate noise. This augmentation is less effective at obscuring sequence at low noise, but at low noise it is more difficult for the model to "ruin" a sample since most of the tertiary organization is in place, and we have good sequences from miniMPNN anyway. This is still our current working hypothesis, but remains an open direction of investigation.

E. Other notes on model development. Initially, we used a convolutional U-Net as the denoiser network and found helical structures to be easy to generate, but beta sheets to be more difficult to properly pair, likely due to their non-local nature. We found that changing the neural network to a U-ViT, removing the convolutional up- and down-sampling layers, and training with self-conditioning (1, 15) all contributed to reducing the frequency of helices and improving the quality of beta sheets. We

Algorithm S1 Denoising step (adapted from "StochasticSampler" (2))

function DENOISINGSTEP($x_t, x_0, \sigma_t, \Delta\sigma, \lambda_{step} = 1, \gamma = (s_{churn}/n_{steps})$)
    $\epsilon \sim \mathcal{N}(\mathbf{0}, \mathbf{I})$
    $\hat{\sigma_t} \leftarrow \sigma_t(1+\gamma)$ ▷ Compute higher noise level
    $\hat{x} \leftarrow x_t + \epsilon\sqrt{\hat{\sigma_t}^2 - \sigma_t^2}$ ▷ Inject noise
    $d \leftarrow (\hat{x} - x_0)/\hat{\sigma_t}$ ▷ Compute gradient $\mathrm{d}\mathbf{x}/\mathrm{d}\sigma$
    $x_{t-1} \leftarrow \hat{x} + \lambda_{step} \cdot \Delta\sigma \cdot d$ ▷ Compute and take Euler step
    output x_{t-1}

also found that network preconditioning and altering the training distribution over noise levels to focus on the most influential noise levels to be important strategies for improving sample quality (2).

When examining more closely some of the early failure modes for sidechain generation, we noticed that most of the sidechains were generated to a reasonable degree of chemical fidelity, but a small percentage of sidechains showed some divergence during sampling, producing bond lengths that were wrong by several angstroms. We hypothesized this was because the sidechains that were infrequently selected during sampling had either too few denoising steps overall, or perhaps a single denoising step that was too large. We did not find a significant association between the largest denoising step and sidechain divergence, but we did note that chemically invalid sidechains seemed to be sampled less frequently on average. To resolve this issue, we annealed the sequence resampling rate with time to increase the rate at which likely sidechains were selected, resampling less and less frequently with time to give these sidechains more denoising steps. This improved the overall quality of the generated sidechains and structures.

F. Sampling and evaluation. For sampling, we used an adapted form of the "stochastic sampler" from Karras et al.(2), which integrates the ODE while optionally injecting noise at each step. Informally, this is done by first stepping to a higher noise level by adding some noise, and then taking a larger denoising step to remove the added noise in addition to the original step size. We make a few adaptations to the algorithm. We do not compute the second order correction since it has only a marginal and inconsistent effect empirically (Supp. Table S1) and doubles the number of neural network calls. We added a step scale which scales the score; this can be interpreted as an inverse temperature parameter or a way to increase the signal amplification which is described and explored in other settings (16–20). We noticed that continually increasing sample quality with s_churn, so we opted not to cap the gamma scale which determines the scale of the noise increase. We describe a subset of experiments we performed to optimize the sampling hyperparameters in Supp. Table S1-2; the most relevant hyperparameters to tune were the number of steps, the amount of churn ("s_churn"), and the step scale.

When running the miniMPNN sequence co-design, we sample from the categorical distribution over amino acids independently for each residue and disallow sampling Cys, Unk, and mask tokens. We find that applying transformations on the logits such as temperature or top-p truncation has little benefit on prediction quality in this scheme. Skipping the early portion of the sampling process has little effect on the sequence quality since the structures are so noisy, and it also increases sampling speed significantly; by default we skip miniMPNN co-design for the first 60% of sampling trajectory steps and use amino acid types sampled uniformly at random. We also found that not resampling the sequence at every step aids in improving sidechain chemical fidelity, and we used a scheme to linearly anneal the resampling rate from 1 to 0 over the portion of the sampling trajectory where we run miniMPNN co-design.

Definitions of metrics computed are given below. We also include non-cherry-picked samples in Supp. Fig. S14-15.

- **Self-consistency.** All self-consistency metrics described are computed with ESMFold (21), and ProteinMPNN for sequence design (22). Typically 8 ProteinMPNN sequences are used for the backbone-only samples (keeping the best by scRMSD) and 1 sequence is used for the all-atom (the one outputted with sampling). For the model comparisons in Table S3, the "short" proteins are run with 8 sequences and the "long" proteins are run with 16 sequences. The scRMSD is computed on alpha-carbon atoms with the Kabsch alignment, and the scTM with TMalign directly.

- **Secondary structure.** Secondary structure is computed with DSSP (using Bio.PDB and the mkdssp package installed with conda).

- **Bond RMSE.** Bond RMSE is computed as the RMSE of every bond from an ideal value for its type (e.g. CA-CB), averaged over all bonds in a structure.

- **Diversity.** Diversity is measured by computing the full matrix of pairwise TM scores for a set of samples, and then taking the mean value of this matrix excluding the diagonal. To compute the filtered diversity metric, we first remove elements from this matrix for which the scTM is below a certain threshold (0.8 by default).

- **Novelty.** Novelty is computed by running US-align to search against the full training set (from https://zhanggroup.org/US-align/ (23)), which runs TMalign under the hood, and reporting the maximum TM score against any training set member. We normalize the TM score by the query (model sample) as recommended by US-align. For the AFDB-trained model we still search against the CATH training set since it would be prohibitively expensive to search against AFDB, and CATH still provides good coverage of possible structural domains.

- **Likelihoods.** Log-likelihoods are computed by solving the probability flow ODE and using the Skilling-Hutchinson trace estimator to compute the vector-Jacobian product as in (24). We use Euler integration with 100 discretization steps and integrate from $\sigma_{min} = 0.01$ to $\sigma_{max} = 800$, and use a standard Gaussian as noise distribution for the trace estimator. We report log-likelihoods in nats/atom, which is the log-likelihood computed in base e averaged over the number of atoms (higher is better), is analogous to bits/dim (equivalent up to a constant), and is also the unit used by Chroma to measure the ELBO (20).

- **Scaffolding success rates.** Scaffolded designs (1 ProteinMPNN sequence) are predicted with ESMFold and we compute the usual self-consistency metrics. The motif RMSD metrics are computed after using TMalign to align the predicted and designed structures. We compute an all-atom motif RMSD which is the RMSD on all atoms (sidechain and backbone) which were conditioned on, as well as a backbone motif RMSD which is restricted to backbone atoms only. Successes are defined as all-atom motif RMSD < 2, backbone motif RMSD < 1, scRMSD < 2, and pLDDT > 70. Weak successes are defined as all-atom motif RMSD < 4, backbone motif RMSD < 3, scTM > 0.5. For the sidechain end/tip atom scaffolding in Fig. 5A (right), we used only the following atoms for each amino acid type as conditioning: "ALA": ["CA", "CB"], "ARG": ["CD", "CZ", "NE", "NH1", "NH2"], "ASP": ["CB", "CG", "OD1", "OD2"], "ASN": ["CB", "CG", "ND2", "OD1"], "CYS": ["CA", "CB", "SG"], "GLU": ["CG", "CD", "OE1", "OE2"], "GLN": ["CG", "CD", "NE2", "OE1"], "GLY": [], "HIS": ["CB", "CG", "CD2", "CE1", "ND1", "NE2"], "ILE": ["CB", "CG1", "CG2", "CD1"], "LEU": ["CB", "CG", "CD1", "CD2"], "LYS": ["CE", "NZ"], "MET": ["CG", "CE", "SD"], "PHE": ["CB", "CG", "CD1", "CD2", "CE1", "CE2", "CZ"], "PRO": ["CA", "CB", "CG", "CD", "N"], "SER": ["CA", "CB", "OG"], "THR": ["CA", "CB", "CG2", "OG1"], "TRP": ["CB", "CG", "CD1", "CD2", "CE2", "CE3", "CZ2", "CZ3", "CH2", "NE1"], "TYR": ["CB", "CG", "CD1", "CD2", "CE1", "CE2", "CZ", "OH"], "VAL": ["CB", "CG1", "CG2"].

G. Conditional generation. We evaluate scaffolding performance on the scaffolding benchmark defined in (25), which is a set of scaffolding tasks based on extracting secondary structure motifs from natural proteins. We remove three tasks for which the contig string is inconsistent with the PDB numbering (6EXZ), and add four tasks based on discontiguous scaffolding for ketosteroid isomerase (PDB ID 1QJG) and de novo enzymes Kemp eliminase (PDB ID 5AOU) and retroaldolase (PDB ID 7K4V), and a version of the 1BCF task which includes only the residues involved in metal binding. We use the full benchmark when evaluating on all-atom conditional generation, and filter to tasks in which all sidechains are fixed for sidechain-conditional generation, since fixing backbones atoms only is meaningless in this context. Additionally, for sidechain-conditional generation we only condition on atoms after the final rotatable bond (as opposed to the full sidechain). While standard replacement guidance was sufficient for conditioning tasks with strong input information, such as inpainting (e.g. benchmark task 2KL8), we found that for tasks such as motif scaffolding with much less input information, the model was prone to ignoring the conditioning during generation. For these tasks, reconstruction guidance was needed to improve the coherence of generated designs with the provided conditioning information (26). When applying reconstruction guidance to sampling we normalize the conditional score to have the same magnitude as the unconditional score and then weight each according to the chosen guidance weight, roughly as λ as $x_{t-1} = x_t + \beta * (\nabla_x \log p(\mathbf{x}) + \lambda \nabla_x \log p(\mathbf{x}|c))/(1+\lambda)$, where β is the step scale. In general the application of step scale and guidance while practically effective does not produce samples from the properly composed or reweighted diffusion distributions, and it may be necessary to apply annealed MCMC (e.g., equilibration with Langevin dynamics) to drive the sampling distributions back to the correct marginals (20, 27). We do so for the conditional generation case and find that sampling from the Langevin dynamics with a Metropolis adjustment (i.e., using the MALA sampler (28)), we are able to enrich for higher scaffolding success rates. Concretely, a single MCMC step involves adding some and removing it with the denoiser, and accepting or rejecting this step with some probability depending on the change in energy (which we define as the time-weighted MSE between the predicted and target motif coordinates).

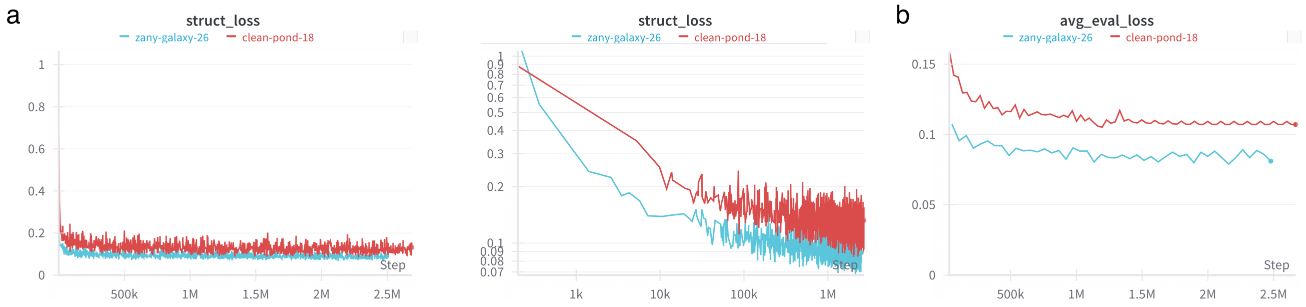

Fig. S1. Loss curves. (a) Training denoising MSE loss curves for backbone-only Protpardelle (blue) and all-atom Protpardelle (red). Both plots show the same data but on different axes scales (right is log-log). **(b)** Validation set denoising MSE loss curves for the same training run. These average loss values are obtained by measuring the MSE on a batch at five different noise levels (t=0.1, 0.3, 0.5, 0.7, 0.9) and averaging over them.

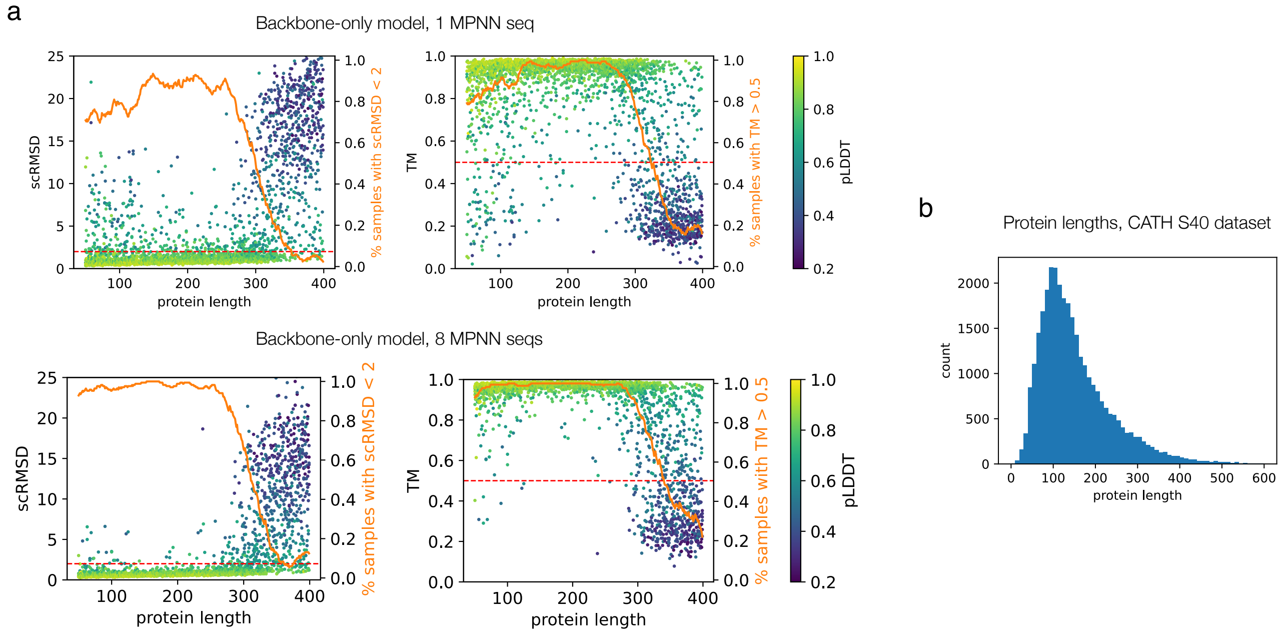

Fig. S2. Additional metrics. (a) Self-consistency metrics for backbone samples, with 1 ProteinMPNN-designed sequence per backbone (top) or 8 sequences (bottom). These are for samples drawn from an older backbone model checkpoint. **(b)** Distribution of protein lengths in the dataset (excluding proteins with length > 600).

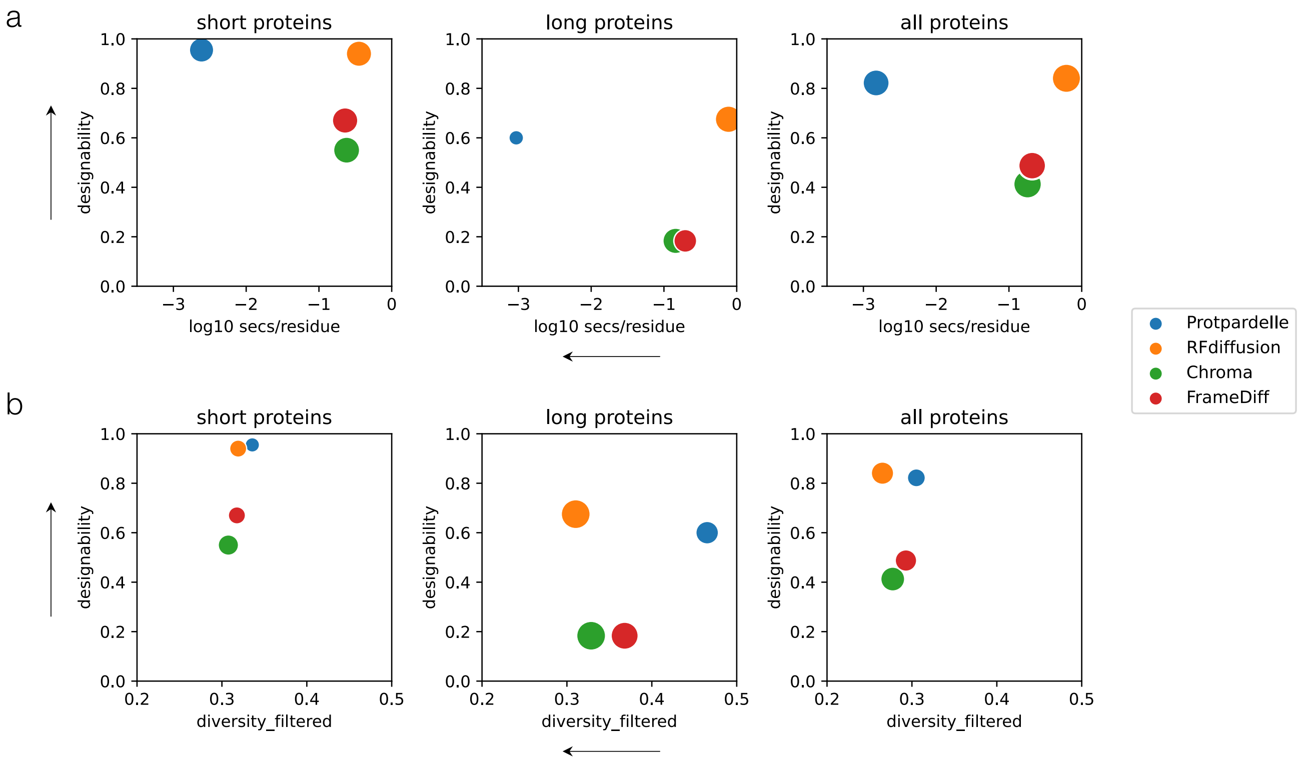

Fig. S3. Comparison to other backbone diffusion methods. Data is taken directly from Supp. Table S3. Short proteins are of length 50-200; long proteins are of length 200-500. **(a)** Designability against sampling time on NVIDIA A40 GPU. Sizes are the diversity on samples with scTM > 0.8; larger is more diverse. A unit step on the x-axis corresponds to a 10-fold speedup. Note: Chroma runtimes include sequence design and packing; excluding these reduces runtimes by 5-10%. **(b)** Designability against diversity on samples with scTM > 0.8. Sizes are the novelty; larger is more novel.

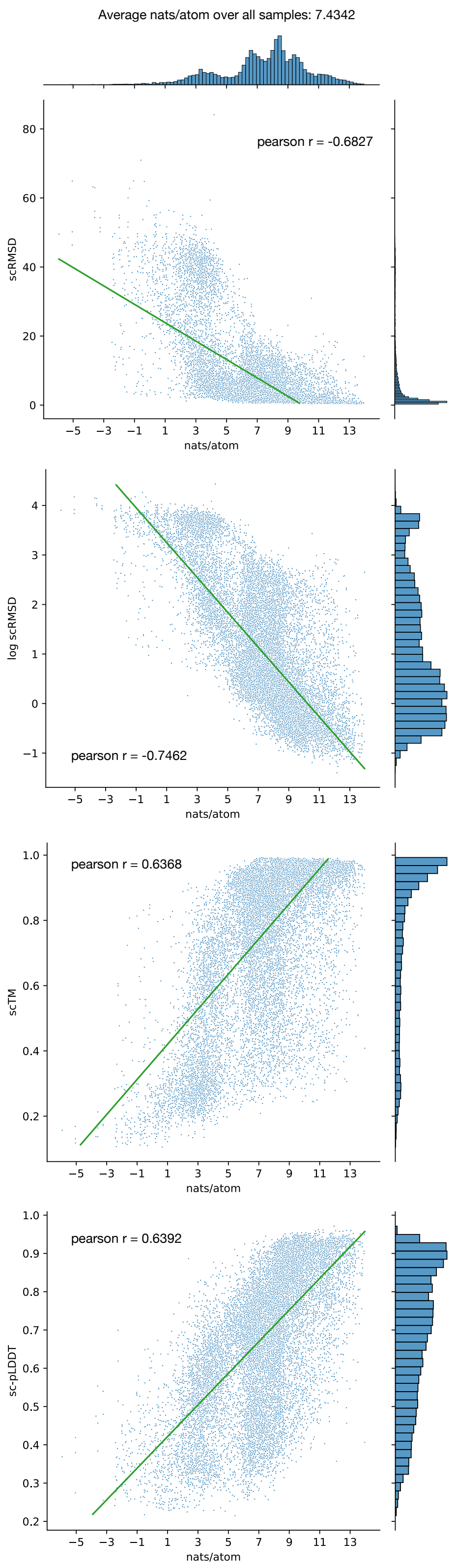

Fig. S4. Correlation of backbone model likelihoods with sample quality metrics (scRMSD, log scRMSD, scTM, and pLDDT). Data are protein structures assembled from various hyperparameter sweeps and evaluation runs for both the backbone-only and all-atom models which have associated self-consistency metrics computed. Note that for all-atom samples, we still compute likelihoods on the backbone structure only.

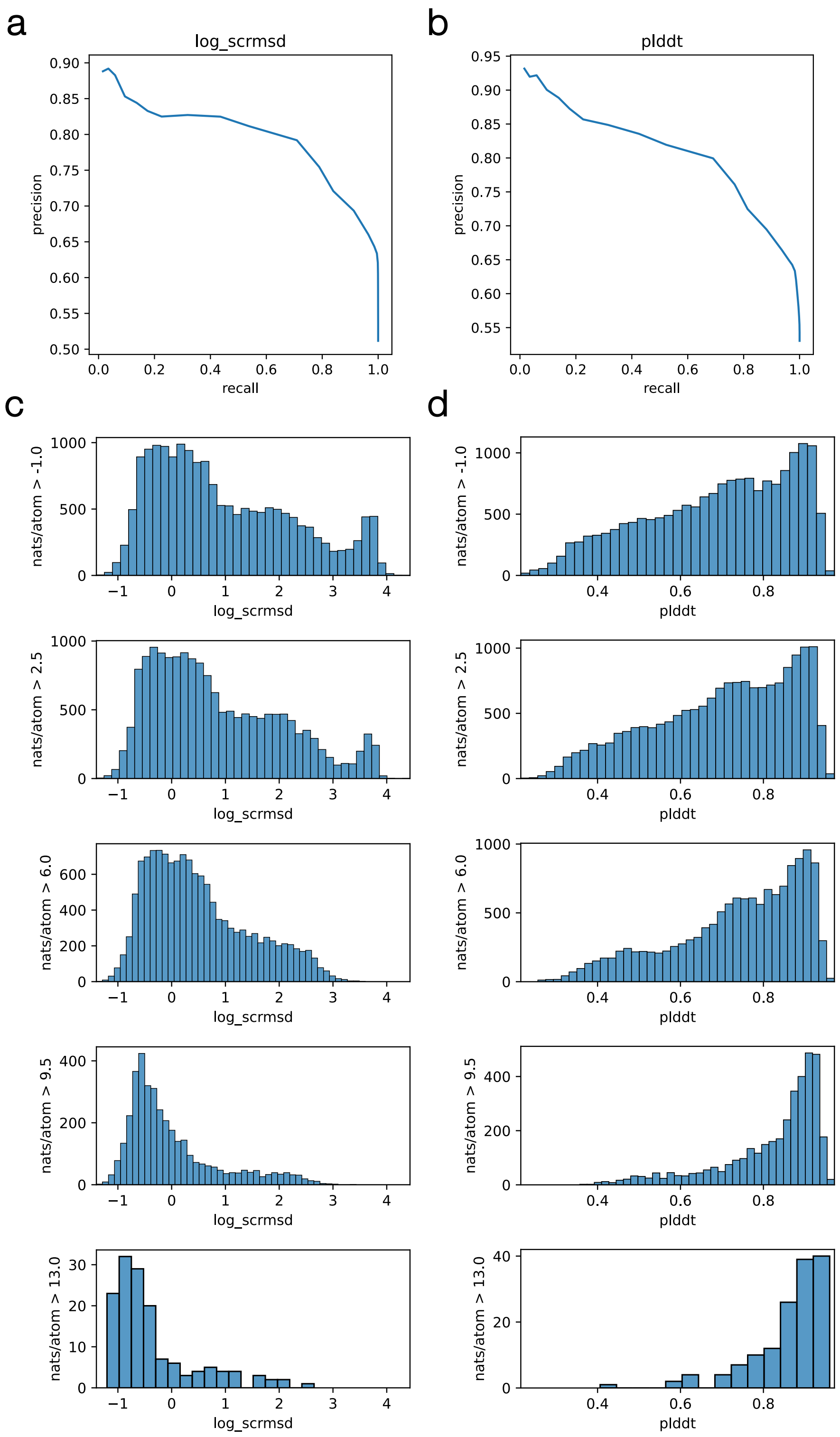

Fig. S5. Viability of model likelihoods as a filtering metric. Data is the same as used in Supp. Fig. S4. **(a-b)** Precision-recall curves, treating model likelihoods as a predictive classifier ("success/positive" is nats/atom greater than some threshold), and using the sample quality metrics as labels ("success/positive" is metric is better than some threshold). For both curves, we scan over likelihood thresholds uniformly from the minimum nats/atom value (approx. -5) to the maximum minus 1 (approx. 13), in order to compute the curve. **(a)** Evaluating ability of likelihood cutoffs to classify scRMSD successes (the curve is the same for scRMSD and log scRMSD, but the distributions in (c) are clearer for log scRMSD). We define a positive label as log-scRMSD < ln 2 = 0.693. **(a)** Evaluating ability of likelihood cutoffs to classify pLDDT successes. We define a positive label as pLDDT > 0.7. **(c-d)** Distributions of **(c)** log scRMSD and **(d)** sc-pLDDT when samples failing different likelihood thresholds are discarded.

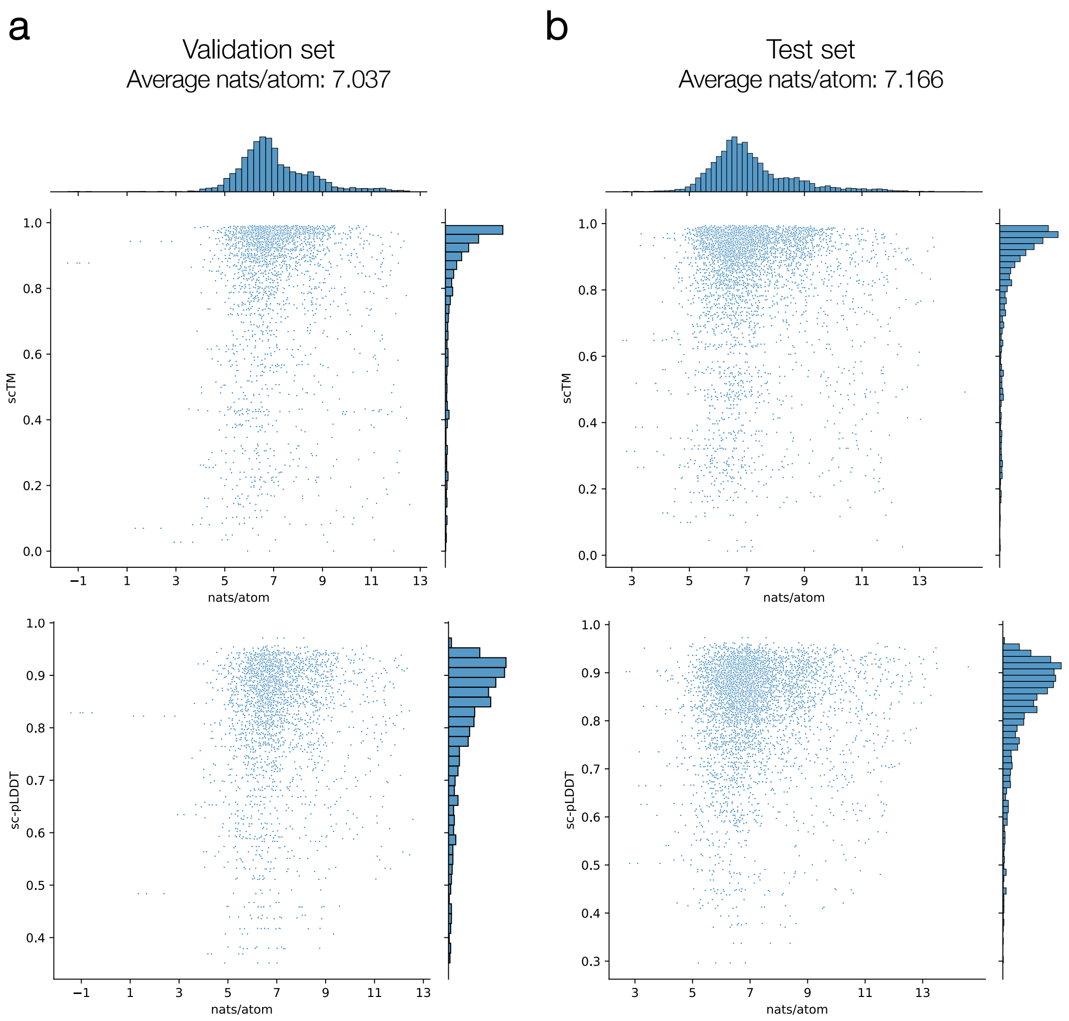

Fig. S6. Model likelihoods on CATH validation and test sets from (7). Likelihoods were computed by backbone-only Protpardelle. Self-consistency metrics were computed by taking the best from 8 ProteinMPNN sequences and their ESMfold predictions.

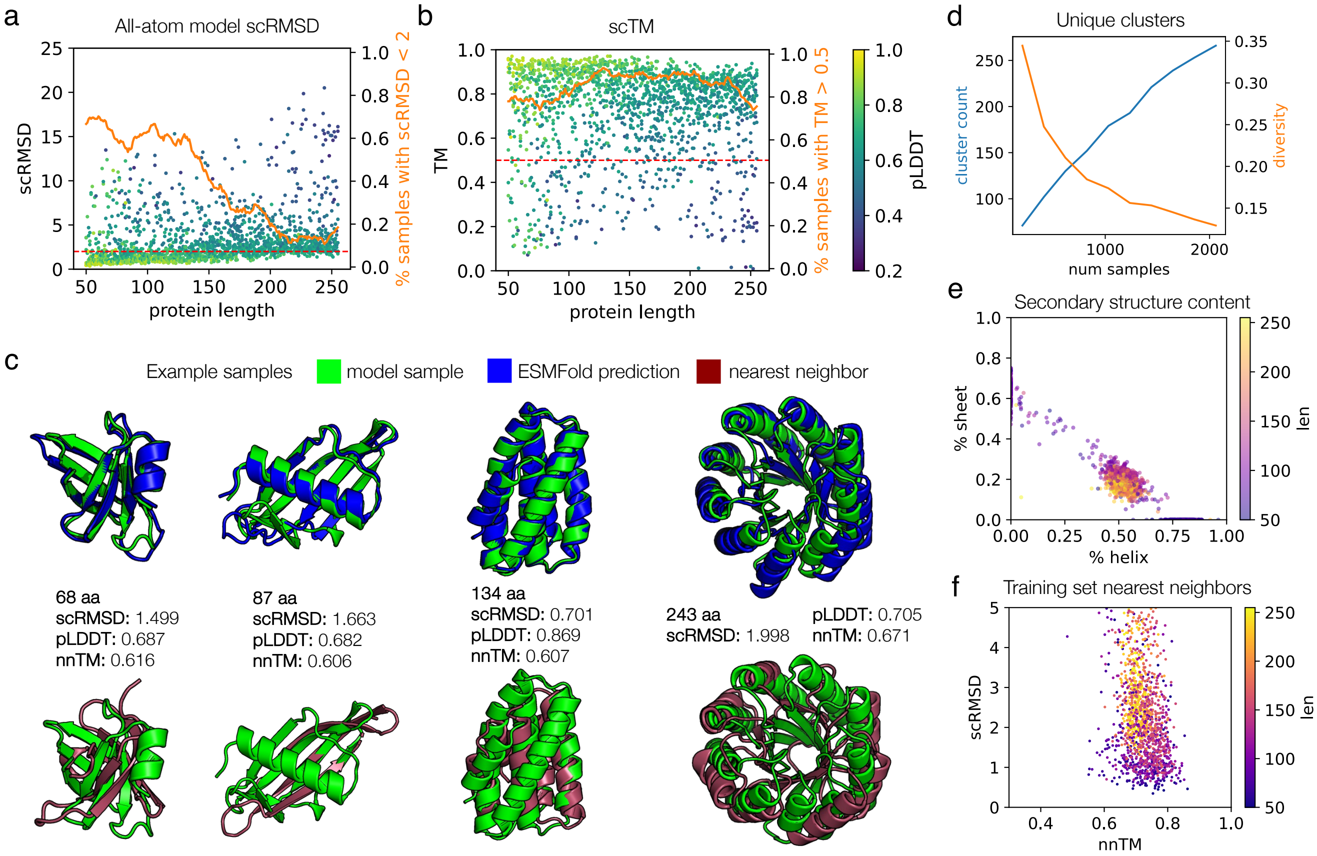

Fig. S7. Evaluation of proteins sampled from the CATH-only all-atom model. (a) Self-consistency performance computed as in Fig. 1, but for the all-atom model. Eight proteins were sampled for each length from 50 to 256. Each protein's sequence is used for ESMFold, i.e. only one sequence is predicted for each sample, rather than the 8 sequences per sample that were predicted for the backbone-only model samples. The success proportion line is smoothed with a sliding window of 21. **(b)** The same samples and ESMFold predictions as in (a), but using the scTM metric. **(c)** Example high-quality, novel all-atom model samples. **(d)** Number of structure clusters per samples drawn (left axis), and ratio of number of clusters to number of samples (right axis). Structures are clustered with MaxCluster as in (29). Samples are drawn uniformly over each length from 50 to 256, as in Fig. 2. **(e)** Secondary structure content of samples, computed by DSSP. **(f)** Nearest neighbor distances for model samples with scRMSD < 5. The nnTM is the TM score against the dataset member with the highest TM score to the sample, extracted with Foldseek.

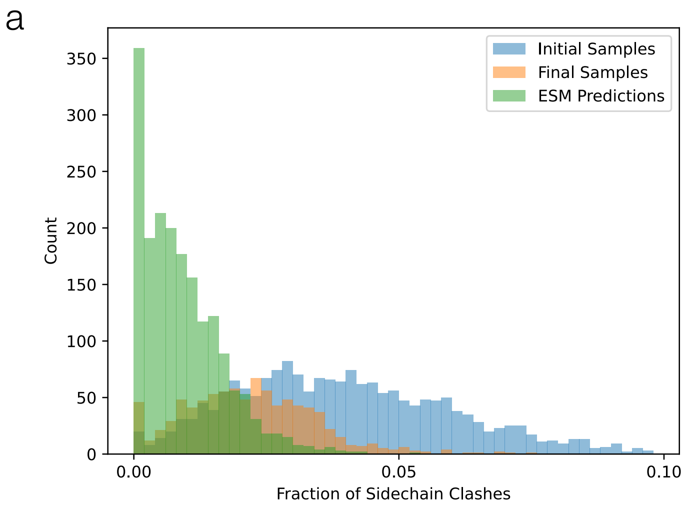

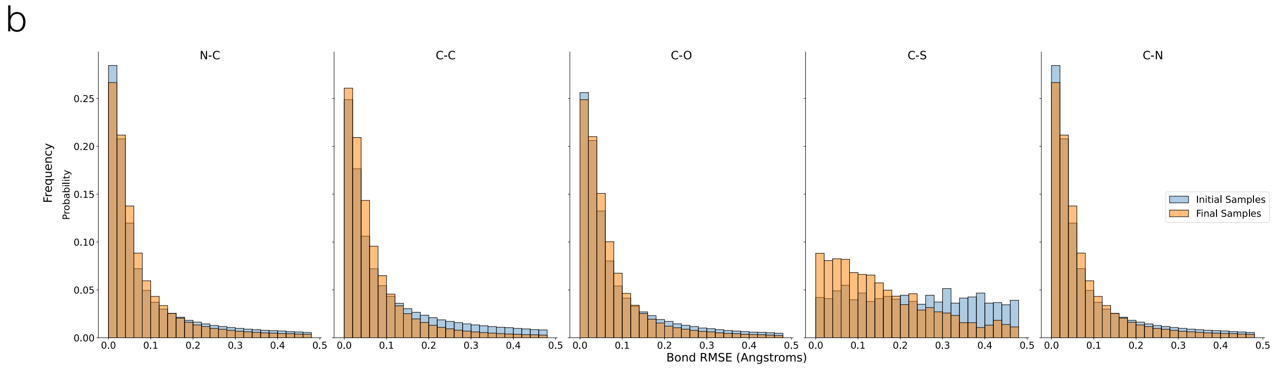

Fig. S8. Two-stage sampling effects on chemical quality. (a) Distributions of the fraction of sidechains involved in a clash, for initial model samples (after stage 1), final model samples (after stage 2), and for the corresponding ESMFold predictions of designed sequences. **(b)** Bond RMSE distributions for both stage 1 and stage 2 (final) samples, faceted by bond type.

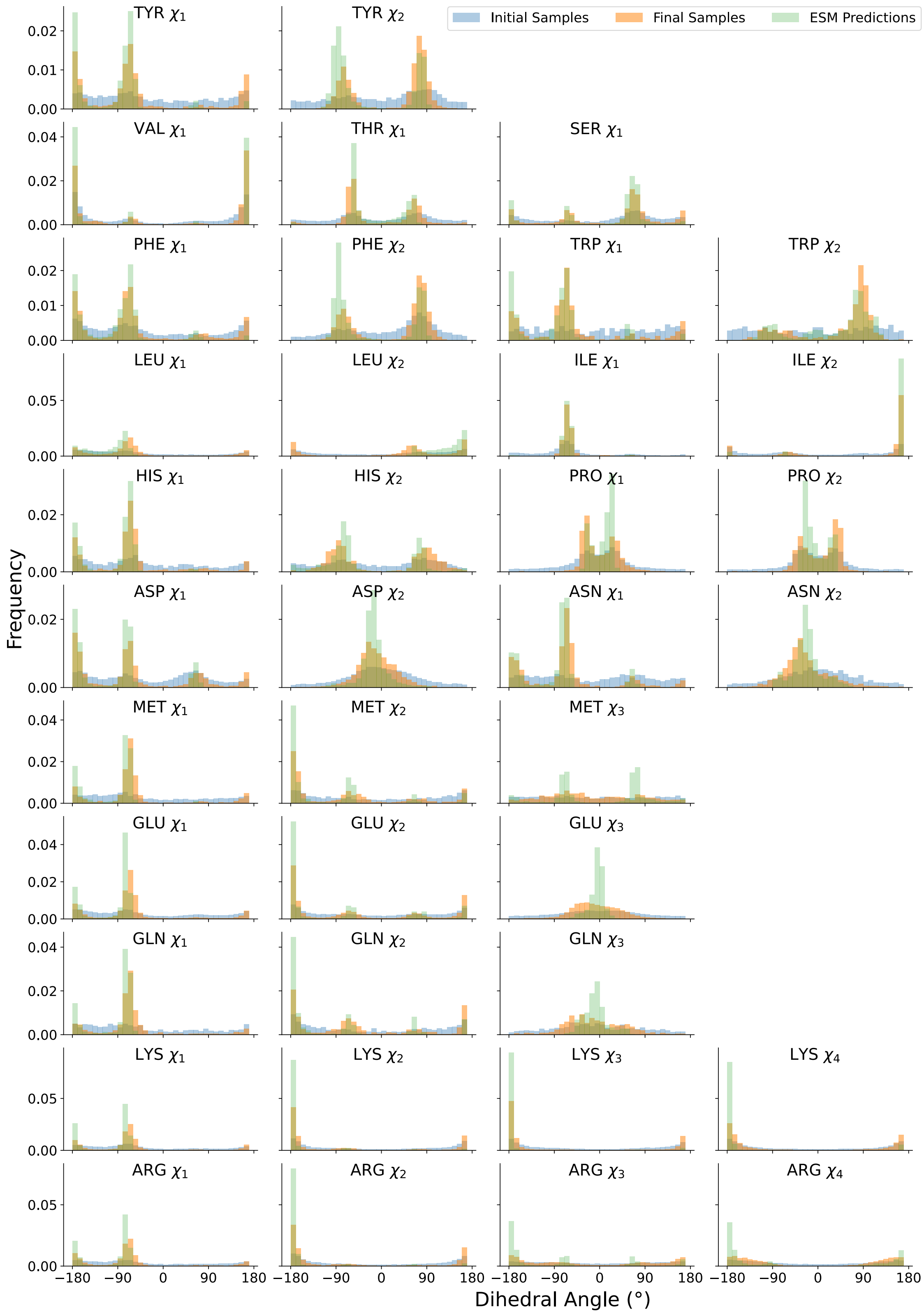

Fig. S9. Chi angle distributions faceted by amino acid type. For each specific chi angle we plot its distribution among stage 1 (initial) samples, stage 2 (final) samples, and the corresponding ESMFold predictions of designed sequences.

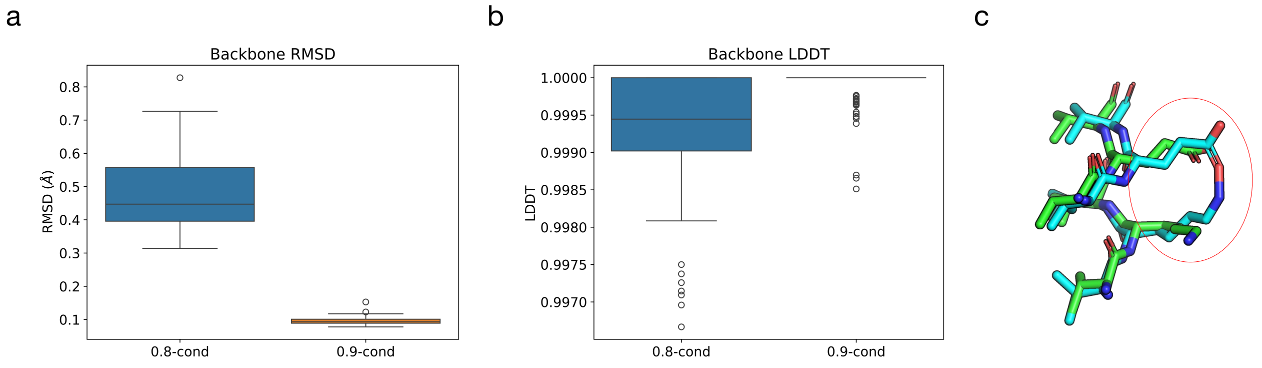

Fig. S10. Backbone adjustment during flexible-bb sidechain repacking. (a) The distribution of backbone RMSD between samples from the 0.8- and 0.9-cond Protpardelle calculations compared to the starting model. Here, "0.9-cond" and "0.8-cond" refer to flexible-backbone sidechain repacking (no sequence change), allowing the entire protein to freely diffuse during the final 10% and 20% of the timesteps, respectively. **(b)** The distribution of backbone LDDT between samples from the 0.8- and 0.9-cond Protpardelle runs compared to the starting models. **(c)** An example of backbone adjustments in response to sidechain repacking. Cyan structure represents a section of the structure from the starting model, originally containing a clash between two sidechains (shown in red circle). Green model represents the same part of the structure from the 0.8-cond Protpardelle calculation. The sidechain clash was resolved with backbone movements.

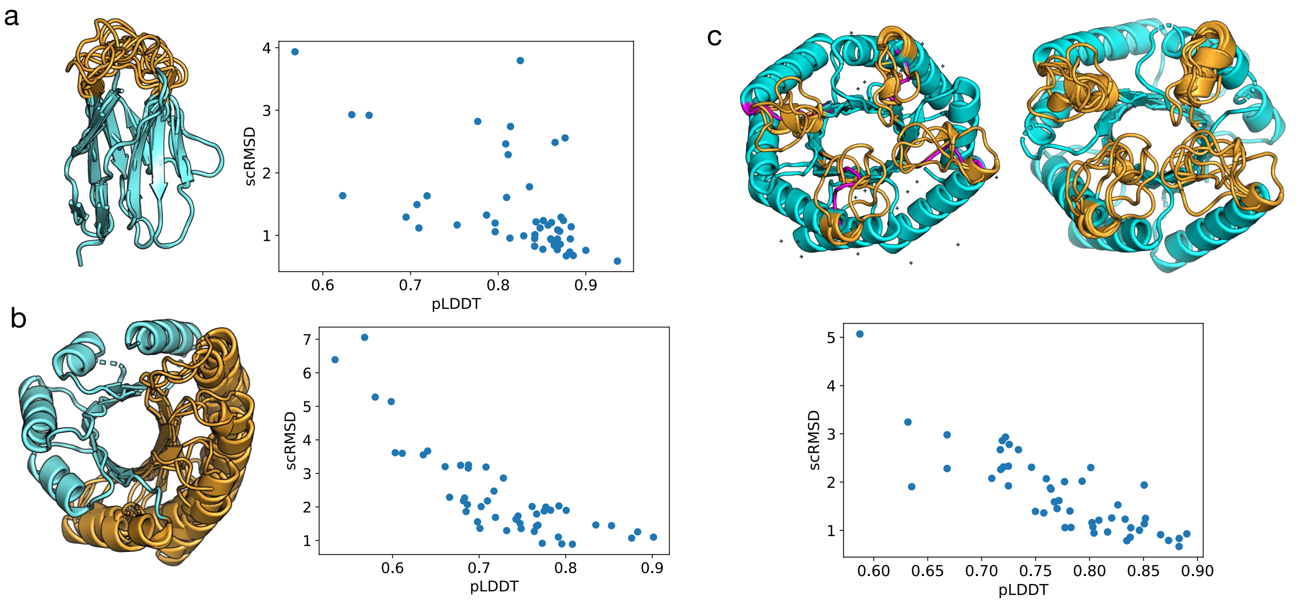

Fig. S11. Inpainting ensembles. Inpainting ensembles for **(a)** a loop of a monobody (PDB: 5X2O, chain L) **(b)** half of a *de novo* TIM barrel (PDB: 5BVL) and **(c)** four loops of a *de novo* ovoid TIM barrel (PDB: 7UEK). The conditioning portion is shown in blue, the model generated portion in brown, and the magenta is the original loop. The inpainted structure in (a) and (b) demonstrate same-length inpainted structure, while the length of the loops in (c) are each four residues longer than the original loop length. Sequences were designed with ProteinMPNN and predicted with ESMFold; design success is shown in the plots.

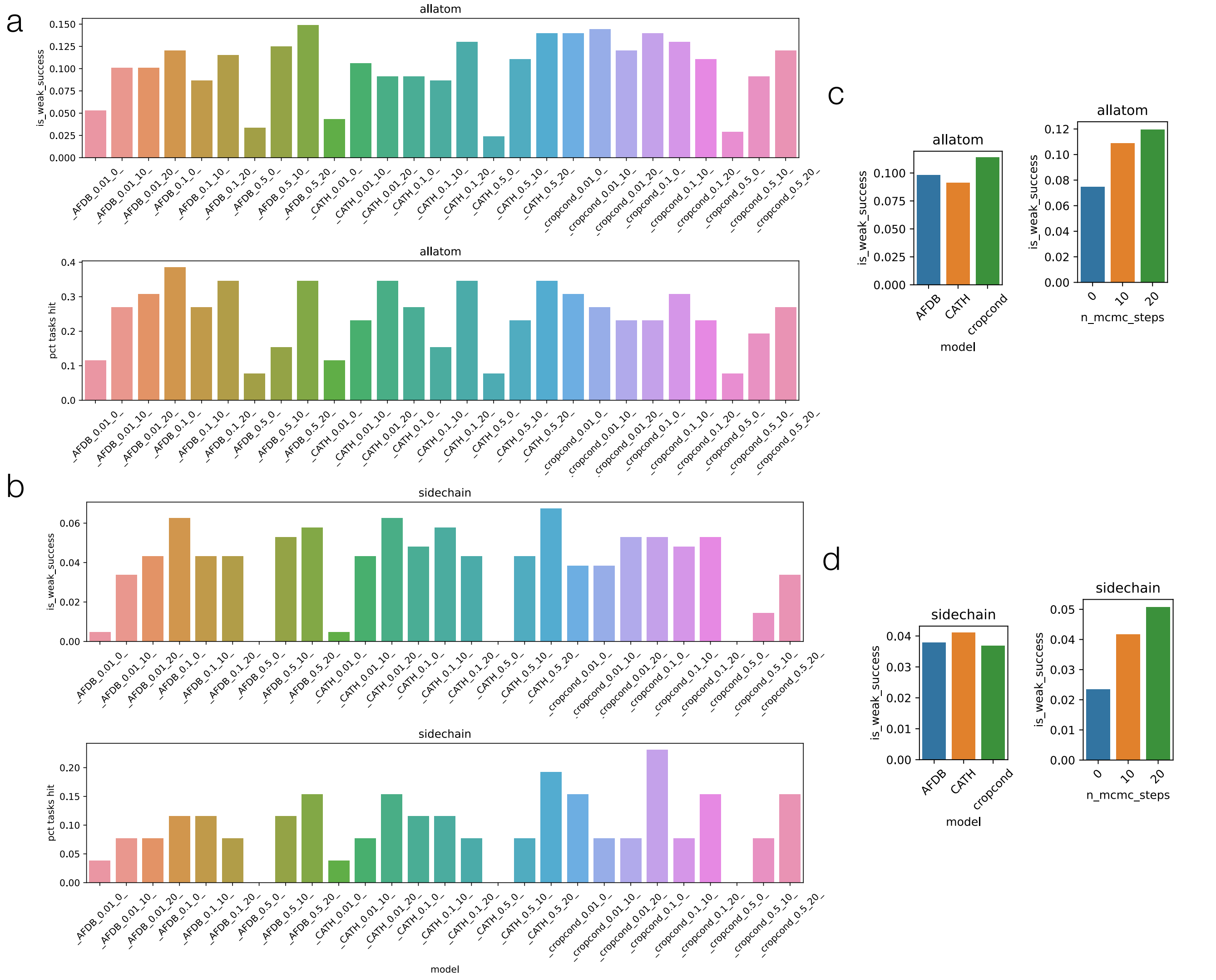

Fig. S12. Effect of experimental parameters on scaffolding performance. (a) The average weak-success rate (top) and percent of tasks with a weak success (bottom) across all-atom scaffolding tasks. Each bar represents and experiment formatted as _[model name]_[guidance scale]_[num MCMC steps]_. CATH refers to the normal all-atom model; AFDB refers to the model trained on CATH and AFDB, and cropcond is a crop-conditional model trained on CATH. **(b)** Same as (a), for sidechain scaffolding tasks. **(c)** The same success-rate data as in (a), aggregated by either the model type or the number of MCMC steps. **(d)** The same success-rate data as in (b), aggregated by either the model type or the number of MCMC steps.

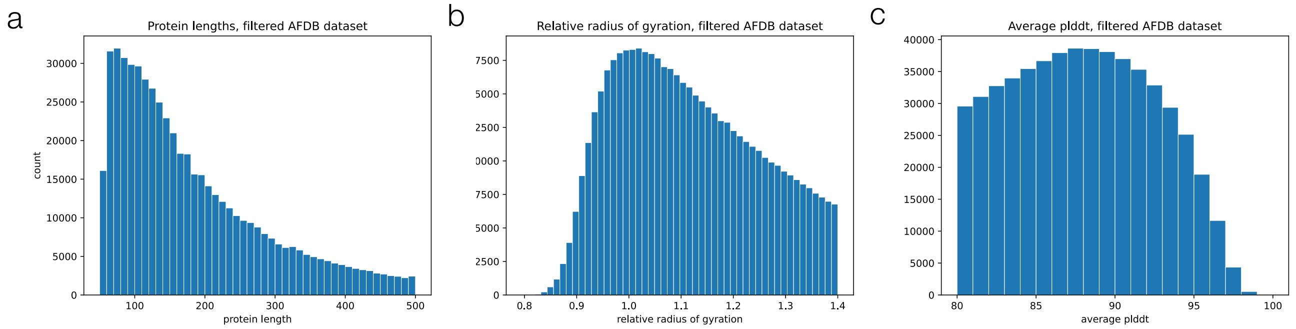

Fig. S13. Statistics for the filtered AFDB dataset. Distributions of **(a)** lengths, **(b)** relative radii of gyration, and **(c)** pLDDTs in the filtered AFDB dataset used to train the all-atom model.

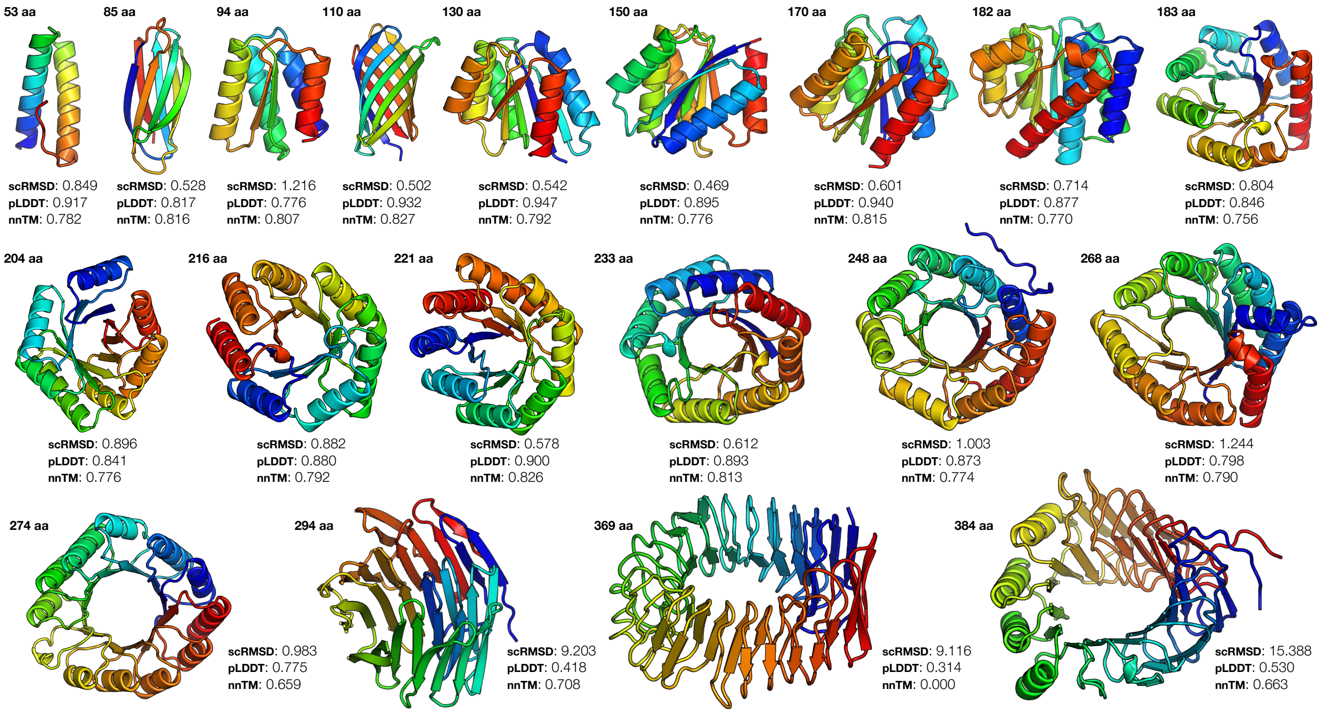

Fig. S14. Samples from backbone Protpardelle. Non-cherry-picked raw samples from the backbone model (older checkpoint). scRMSD is the best out of 8 ProteinMPNN sequences with ESMFold, with the corresponding pLDDT.

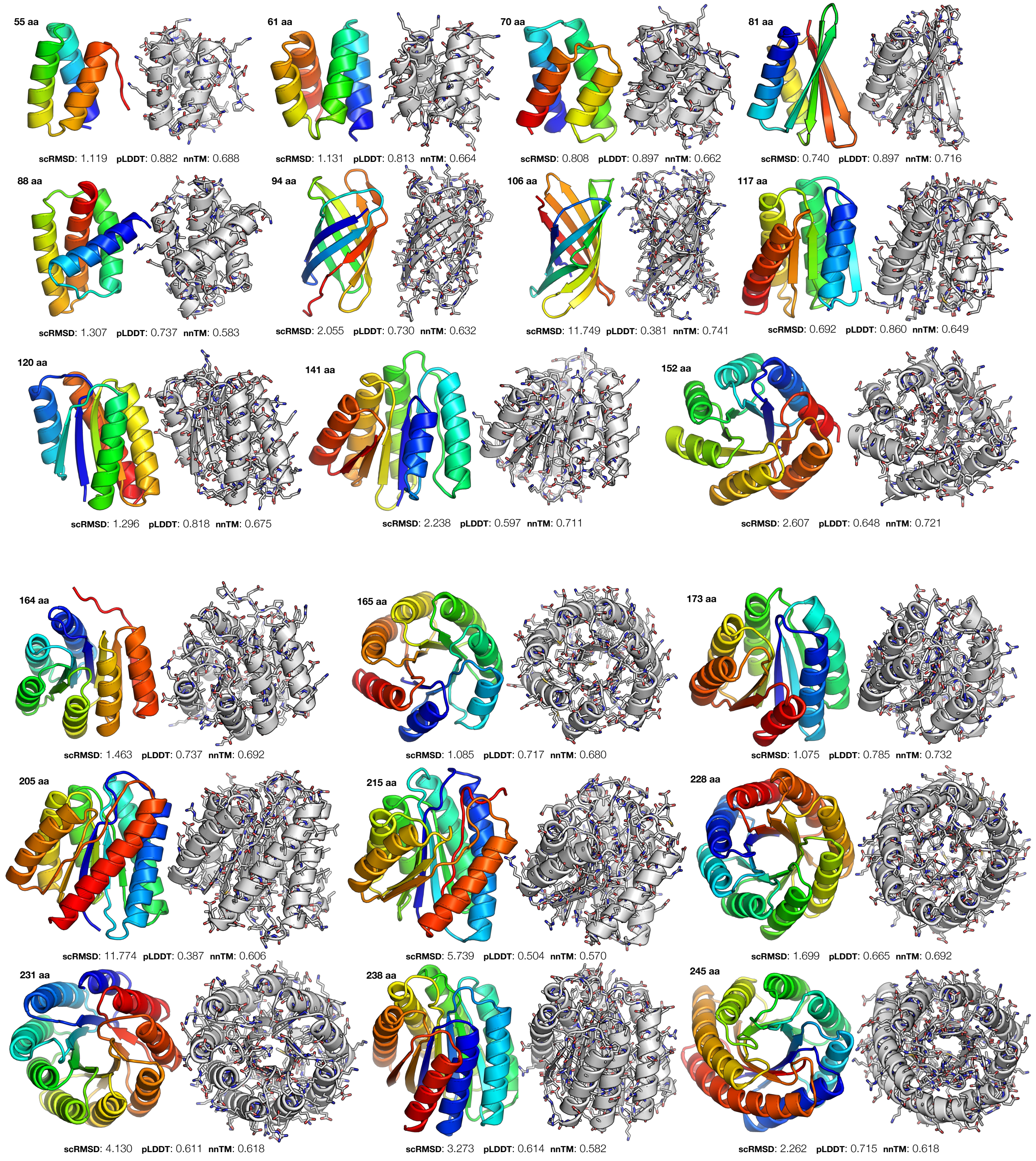

Fig. S15. Samples from all-atom Protpardelle. Non-cherry-picked raw samples from the CATH-only all-atom model. scRMSD is the best out of 8 ProteinMPNN sequences with ESMFold, with the corresponding pLDDT. The structure without sidechains is shown in color, and the same structure with sidechains shown adjacent without color.

Table S1. Backbone model sampling hyperparameter sweeps. For each row here 2 samples in lengths 50:400:10 were sampled, and the best of 8 ProteinMPNN sequences by scRMSD was used. steps: number of ODE discretization steps. schurn: s_churn value (divide by steps to get the scale of additional noise added at each step). step scale: scale applied to Euler step. 2nd order correction: whether the 2nd order correction in the original stochastic sampler was used. designability: percent of samples with scRMSD < 2. diversity: mean pairwise TM score between samples. diversity (filtered): the same metric only on samples with scTM > 0.8. mean scRMSD: mean scRMSD over all samples. median scRMSD: median scRMSD over all samples. mean scTM: mean scTM over all samples. runtime: total sampling time for all samples in seconds on NVIDIA A40 GPU. seconds / 100 residues: runtime divided by the total number of residues generated across all samples.

steps	schurn	step scale	2nd order correction	designability (↑)	diversity (↓)	diversity (filtered) (↓)	mean scRMSD (↓)	median scRMSD (↓)	mean scTM (↑)	runtime (s) (↓)	seconds / 100 residues (↓)
20	1	1.1	False	0.014	0.262		12.837	13.024	0.412	**5.800**	**0.038**
20	1	1.1	True	0.029	**0.250**	0.289	10.197	10.957	0.459	9.702	0.063
50	1	1.1	False	0.243	0.272	**0.283**	6.626	6.068	0.648	62.306	0.405
50	1	1.1	True	0.214	0.268	0.295	6.056	5.833	0.666	71.901	0.467
200	0	1.0	False	0.129	0.274	0.336	5.543	4.301	0.670	93.195	0.605
200	20	1.0	False	0.500	0.300	0.325	2.852	1.985	0.830	106.780	0.693
200	20	1.05	False	0.671	0.313	0.340	1.962	1.523	0.880	94.525	0.614
200	20	1.1	False	0.671	0.320	0.344	1.940	1.540	0.882	43.324	0.281
200	20	1.2	False	0.829	0.331	0.346	1.540	1.142	0.913	43.492	0.282
200	20	1.2	True	0.771	0.328	0.347	1.571	1.176	0.910	80.230	0.521
200	40	1.2	True	0.829	0.321	0.354	1.580	1.025	0.914	81.476	0.529
500	0	1.0	False	0.171	0.278	0.312	4.780	3.968	0.720	150.629	0.978
500	0	1.1	False	0.671	0.317	0.340	2.594	1.488	0.872	172.231	1.118
500	100	1.1	False	0.929	0.337	0.348	1.039	0.820	0.950	150.536	0.978
500	200	1.2	False	**0.986**	0.360	0.360	**0.801**	**0.721**	**0.969**	155.577	1.010
500	200	1.2	True	**0.986**	0.350	0.353	0.978	0.764	0.960	261.598	1.699

Table S2. All-atom model sampling hyperparameter sweeps. Only 1 ProteinMPNN sequence (the one outputted with the sample) is used for each sample. All columns have the same meanings as in Table S1.

steps	schurn	step scale	2nd order correction	designability (↑)	diversity (↓)	diversity (filtered) (↓)	mean scRMSD (↓)	median scRMSD (↓)	mean scTM (↑)	runtime (s) (↓)	seconds / 100 residues (↓)
200	0	1.0	False	0.014	<u>0.262</u>	<u>0.301</u>	10.769	9.049	0.509	341.899	2.220
200	0	1.2	False	0.129	0.327	0.393	8.456	4.871	0.623	**284.190**	**1.845**
200	100	1.0	False	0.129	0.302	0.412	8.803	3.467	0.668	349.772	2.271
200	100	1.2	False	0.057	0.328	0.459	8.755	6.388	0.572	<u>286.049</u>	<u>1.857</u>
500	0	1.0	False	0.043	**0.237**	**0.298**	11.432	7.815	0.529	435.671	2.829
500	0	1.1	False	0.114	0.296	0.390	6.923	4.191	0.677	438.975	2.850
500	0	1.2	False	0.200	0.307	0.420	6.675	3.983	0.682	436.810	2.836
500	100	1.0	False	0.157	0.301	0.459	6.044	2.757	0.729	436.067	2.832
500	100	1.1	False	<u>0.514</u>	0.311	0.415	5.187	1.972	0.786	437.910	2.844
500	100	1.2	False	**0.614**	0.330	0.412	<u>4.347</u>	<u>1.860</u>	<u>0.826</u>	439.644	2.855
500	200	1.2	False	**0.614**	0.334	0.403	**3.526**	**1.719**	**0.836**	442.221	2.872

Table S3. Comparison of backbone only model to other backbone diffusion models. All columns have the same meanings as in Table S1. Total residues is the total number of residues sampled. Short: 4 samples per length in 50:200:3, with 8 ProteinMPNN sequences per sample. Long: 4 samples per length in 200:500:10, with 16 ProteinMPNN sequences per sample. All proteins is the union of these two sets. Protpardelle (500) and Protpardelle (200) are with 500 and 200 steps respectively and sampling hyperparameters as noted. 1x ProteinMPNN indicates only 1 ProteinMPNN sequence used instead of 8 for the Protpardelle (500) samples. w/ AF2 indicates that AF2 instead of ESMFold was used as the structure prediction network for Protpardelle (500). FrameDiff, Chroma, and RFdiffusion were sampled using the default public configurations (20, 25, 29). *Note that Chroma runtimes include sequence design and packing; excluding these reduces runtimes by 5-10%. Also, for consistency we do not use the Chroma-designed sequences for subsequent structure prediction, even though it may not have been optimized specifically for ProteinMPNN. **Protpardelle (200) is the main/default backbone model.

	**steps**	**schurn**	**step scale**	**design-ability (↑)**	**diversity (↓)**	**diversity filtered (↓)**	**novelty (↓)**	**mean scRMSD (↓)**	**median scRMSD (↓)**	**mean scTM (↑)**	**runtime (s) (↓)**	**total residues**	**seconds / 100 residues (↓)**
**Short**													
Protpardelle (500)	500	200	1.2	**0.995**	0.372	0.372	0.708	**0.590**	0.560	**0.970**	201.787	24700	0.817
1x ProteinMPNN	500	200	1.2	0.940	-	-	-	1.074	0.767	0.929	-	-	-
w/ AF2	500	200	1.2	0.705	-	-	-	2.446	1.201	0.841	-	-	-
Protpardelle (200) (main)	200	40	1.2	0.955	0.329	0.336	0.669	0.942	0.755	0.936	**60.172**	24700	**0.244**
FrameDiff	-	-	-	0.670	0.298	0.318	0.641	2.373	1.302	0.825	5650.250	24700	22.876
Chroma	-	-	-	0.550	**0.284**	**0.308**	**0.604**	2.869	1.755	0.793	5928.690	24700	24.003
RFDiffusion	-	-	-	0.940	0.315	0.319	0.642	0.857	**0.552**	0.952	8761.270	24700	35.471
**Long**													
Protpardelle (500)	500	200	1.2	**0.842**	0.435	0.507	0.569	4.078	**0.836**	0.909	160.411	41400	0.387
1x ProteinMPNN	500	200	1.2	0.742	-	-	-	5.923	1.241	0.868	-	-	-
w/ AF2	500	200	1.2	0.183	-	-	-	8.794	5.435	0.674	-	-	-
Protpardelle (200) (main)	200	40	1.2	0.600	0.398	0.465	0.562	3.870	1.550	0.855	**38.655**	41400	**0.093**
FrameDiff	-	-	-	0.183	0.309	0.368	0.490	7.731	6.934	0.657	8130.040	41400	19.638
Chroma	-	-	-	0.183	**0.285**	0.329	0.457	6.645	5.781	0.707	5989.730	41400	14.468
RFDiffusion	-	-	-	0.675	0.307	**0.310**	**0.456**	**2.368**	0.892	**0.910**	31969.300	41400	77.221
**All proteins**													
Protpardelle (500)	500	200	1.2	**0.938**	0.309	0.324	0.656	1.898	0.647	**0.947**	362.198	66100	0.548
1x ProteinMPNN	500	200	1.2	0.866	-	-	-	2.892	0.899	0.906	-	-	-
w/ AF2	500	200	1.2	0.509	-	-	-	4.826	1.954	0.779	-	-	-
Protpardelle (200) (main)	200	40	1.2	0.822	0.285	0.305	0.629	2.040	0.878	0.906	**98.827**	66100	**0.150**
FrameDiff	-	-	-	0.487	0.254	0.293	0.585	4.382	2.119	0.762	13780.290	66100	20.848
Chroma	-	-	-	0.412	**0.241**	0.277	**0.549**	4.285	2.670	0.761	11918.420	66100	18.031
RFDiffusion	-	-	-	0.841	0.259	**0.265**	0.572	**1.423**	**0.626**	0.936	40730.570	66100	61.620

Table S4. Ablations on all-atom model. Columns and "short", "long" and "all" sets have the same meanings as in Table S3. Only 1 ProteinMPNN sequence (the one outputted with the sample) is used for each sample. stage 1 bond RMSE: bond RMSE for initial samples. stage 2 bond RMSE: bond RMSE after sidechain repacking/refinement step. Runtimes include both stage 1 and stage 2 sampling time. default: CATH-only all-atom model. mini MPNN: same as default, but use the miniMPNN predicted sequence rather than ProteinMPNN as the final output sequence. w/ AF2: same as default, but use AF2 instead of ESMFold as the structure prediction network. afdb: model trained on CATH + AFDB.

	**steps**	**schurn**	**step scale**	**design-ability (↑)**	**diversity (↓)**	**diversity (filtered) (↓)**	**novelty (↓)**	**mean scRMSD (↓)**	**median scRMSD (↓)**	**mean scTM (↑)**	**stage 1 bond RMSE (↓)**	**stage 2 bond RMSE (↓)**	**runtime (s) (↓)**	**total residues**	**seconds / 100 residues (↓)**
**Short**															
default	500	200	1.2	**0.800**	0.384	0.399	0.706	**1.851**	**1.259**	**0.863**	0.646	**0.073**	697.799	24700	2.825
mini MPNN	500	200	1.2	0.090	-	-	-	6.548	5.279	0.596	-	-	-	-	-
w/ AF2	500	200	1.2	0.510	-	-	-	3.778	1.960	0.755	-	-	-	-	-
afdb	500	200	1.2	0.730	**0.312**	**0.324**	**0.696**	2.080	1.332	0.849	**0.557**	0.099	**690.146**	24700	**2.794**
**Long**															
default	500	200	1.2	**0.425**	**0.330**	0.586	**0.545**	13.644	**2.402**	0.691	**0.619**	0.138	**667.397**	41400	**1.612**
mini MPNN	500	200	1.2	0.000	-	-	-	20.273	9.942	0.505	-	-	-	-	-
w/ AF2	500	200	1.2	0.042	-	-	-	14.241	9.115	0.569	-	-	-	-	-
afdb	500	200	1.2	0.142	0.464	**0.434**	0.630	**6.284**	5.558	**0.699**	0.758	**0.104**	716.112	41400	1.730
**All proteins**															
default	500	200	1.2	**0.659**	0.294	0.363	**0.646**	6.274	**1.606**	**0.798**	0.679	0.119	**1365.197**	66100	**2.065**
mini MPNN	500	200	1.2	0.056	-	-	-	11.695	6.004	0.562	-	-	-	-	-
w/ AF2	500	200	1.2	0.334	-	-	-	7.702	3.776	0.685	-	-	-	-	-
afdb	500	200	1.2	0.509	**0.286**	**0.307**	0.671	**3.657**	1.945	0.793	**0.666**	**0.104**	1406.258	66100	2.127

Table S5. Comparing sidechain repacking on "good" Protpardelle-designed all-atom samples. We compared different purpose-trained sidechain packing methods against Protpardelle at repacking sidechains on starting (stage1) all-atom Protpardelle samples. We subselected samples with Cα-scRMSD < 1.0 and repacked sidechains, reporting (1) the average percentage of residues per protein whose sidechains clash with the sidechain or the backbone of a different residue (Total Clashes) (2) the average percentage of residues per protein whose sidechains clash with only the sidechain of a different residue (Sidechain Clashes), as described in (30). For "fixed-backbone Protpardelle" repacking, we fixed the backbone and sequence of the stage1 sample. For 0.9-cond and 0.8-cond Protpardelle, we allowed the backbone to freely diffuse during the final 10% and 20% of the timesteps, respectively, to enable backbone adjustment. We compare Protpardelle repacking with the RosettaPacker (31), AttnPacker (32), and Chroma (20).

Dataset	Total Clashes	Sidechain Clashes
Protpardelle Stage 1	9.03%	4.60%
RosettaPacker	0.82%	0.06%
AttnPacker	1.27%	0.38%
Chroma (sc-packing)	1.88%	0.92%
Fixed-bb Protpardelle	5.44%	2.47%
0.9-cond Protpardelle	4.94%	2.38%
0.8-cond Protpardelle	3.78%	2.19%

Table S6. Comparing sidechain packing on "good" ESMFold-generated samples. The same analysis as in Table S5, but using the ESMFold predicted structures of the selected samples in Table S5, which might contain fewer backbone abnormalities compared with the raw samples. "ESMFold" indicates the baseline clash rate in the output ESMFold prediction. We compare Protpardelle repacking with the RosettaPacker (31), AttnPacker (32), Chroma (20) and ESMFold (21).

Dataset	Total Clashes	Sidechain Clashes
ESMFold	0.80%	0.62%
RosettaPacker	0.11%	0.04%
AttnPacker	0.06%	0.00%
Chroma (sc-packing)	0.87%	0.63%
Fixed-bb Protpardelle	5.64%	2.58%
0.9-cond Protpardelle	5.56%	2.71%
0.8-cond Protpardelle	4.71%	2.68%

Table S7. Sampling hyperparameter sweeps for sidechain repacking. We compared total clashes and sidechain clashes with the protpardelle stage1 sample used in Table S5, changing sampling hyperparameters for sidechain repacking (second stage sampling). reconstruction guidance: whether the reconstruction guidance is used. replacement guidance: whether the replacement guidance is used. guidance scale: scale for reconstruction guidance. All sampling used steps 200, schurn 100, step scale 1.0, without 2nd order correction.

Replacement Guidance	Reconstruction Guidance	Guidance Scale	0.8-cond Total Clashes	0.9-cond Total Clashes	Fixed-bb Total Clashes	0.8-cond Sidechain Clashes	0.9-cond Sidechain Clashes	Fixed-bb Sidechain Clashes
True	False	-	3.84%	5.96%	5.32%	2.43%	2.40%	2.25%
True	True	0.01	4.02%	5.35%	5.13%	2.61%	2.35%	2.20%
True	True	0.03	4.32%	4.65%	4.94%	2.58%	2.14%	2.26%
True	True	0.05	**3.78%**	4.94%	5.44%	**2.19%**	2.38%	2.47%
True	True	0.07	3.80%	5.10%	5.00%	2.26%	2.25%	2.29%
True	True	0.09	4.01%	4.90%	5.00%	2.36%	2.21%	2.23%

Table S8. Comparing sidechain packing on CASP13 proteins. The MAE of each dihedral angle reported in degrees and the percentage of sidechain clashes for the public CASP13 target proteins. The average percentage of residues per protein whose sidechains clash with the sidechain of a different residue as described and calculated by Oxford Protein Informatics Group (30). The CASP13 target proteins used in this set are: T0950, T0951, T0953s1, T0953s2, T0954, T0955, T0957s1, T0957s2, T0958, T0960, T0963, T0966, T0968s1, T0968s2, T1003, T1005, T1008, T1009, T1011, T1016. We compare fixed-backbone, fixed-sequence Protpardelle with RosettaPacker (31), AttnPacker (32), and Chroma (20).

Dataset	MAE (°)				Sidechain Clashes
	χ_1	χ_2	χ_3	χ_4	
RosettaPacker	16.38	22.22	36.90	39.16	0.00%
AttnPacker	1.67	8.72	22.09	2.87	0.00%
Chroma (sc-packing)	12.40	18.02	34.87	32.68	0.17%
Fixed-bb Protpardelle	40.70	39.11	46.82	43.75	6.37%

Table S9. Comparing sidechain packing on CASP14 proteins The MAE of each dihedral angle reported in degrees and the percentage of sidechain clashes for the public CASP14 target proteins. The average percentage of residues per protein whose sidechains clash with the sidechain of a different residue as described and calculated by Oxford Protein Informatics Group (30). The CASP14 target proteins used in this set are: T1024, T1025, T1026, T1027, T1029, T1030, T1032, T1033, T1035, T1036s1, T1037, T1038, T1039, T1040, T1041, T1042, T1043, T1044, T1046s1, T1046s2, T1049, T1050, T1054, T1056, T1064, T1067, T1073, T0174, T1079, T1080, T1082, T1090, T1099. We compare fixed-backbone, fixed-sequence Protpardelle with RosettaPacker (31), AttnPacker (32), and Chroma (20).

**Dataset**	**MAE** (°)				**Sidechain Clashes**
	χ_1	χ_2	χ_3	χ_4	
RosettaPacker	29.22	35.22	47.96	46.36	0.00%
AttnPacker	8.27	18.11	26.63	8.08	0.00%
Chroma (sc-packing)	18.95	23.94	42.58	39.20	0.46%
Fixed-bb Protpardelle	46.27	45.66	48.75	42.88	6.03%

References

1. E Hoogeboom, J Heek, T Salimans, simple diffusion: End-to-end diffusion for high resolution images (2023).
2. T Karras, M Aittala, T Aila, S Laine, Elucidating the design space of diffusion-based generative models (2022).
3. E Perez, F Strub, H de Vries, V Dumoulin, A Courville, Film: Visual reasoning with a general conditioning layer (2017).
4. W Peebles, S Xie, Scalable diffusion models with transformers (2023).
5. Z Zheng, et al., Structure-informed language models are protein designers (2023).
6. NL Dawson, et al., Cath: An expanded resource to predict protein function through structure and sequence. *Nucleic Acids Res.* **45** (2016).
7. J Ingraham, VK Garg, R Barzilay, T Jaakkola, Generative models for graph-based protein design in *Advances in Neural Information Processing Systems.* (2019).
8. D Kingma, J Ba, Adam: A method for stochastic optimization. *Int. Conf. on Learn. Represent.* (2014).
9. T Chen, On the importance of noise scheduling for diffusion models (2023).
10. M Varadi, et al., AlphaFold Protein Structure Database: massively expanding the structural coverage of protein-sequence space with high-accuracy models. *Nucleic Acids Res.* **50**, D439–D444 (2021).
11. M van Kempen, et al., Fast and accurate protein structure search with foldseek. *Nat. Biotechnol.* (2023).
12. I Barrio-Hernandez, et al., Clustering predicted structures at the scale of the known protein universe. *Nature* **622**, 637–645 (2023) Number: 7983 Publisher: Nature Publishing Group.
13. R Verkuil, et al., Language models generalize beyond natural proteins. *bioRxiv* (2022).
14. KA Dill, K Ghosh, JD Schmit, Physical limits of cells and proteomes. *Proc. Natl. Acad. Sci.* **108**, 17876–17882 (2011) Publisher: Proceedings of the National Academy of Sciences.
15. T Chen, R Zhang, G Hinton, Analog bits: Generating discrete data using diffusion models with self-conditioning (2023).
16. P Dhariwal, A Nichol, Diffusion models beat gans on image synthesis (2021).
17. J Ho, T Salimans, Classifier-free diffusion guidance (2022).
18. J Ho, et al., Imagen video: High definition video generation with diffusion models (2022).
19. R Aditya, et al., Zero-shot text-to-image generation (2021).
20. J Ingraham, et al., Illuminating protein space with a programmable generative model. *Nature* **623**, 1070–1078 (2023).
21. Z Lin, et al., Evolutionary-scale prediction of atomic-level protein structure with a language model. *Science* **379**, 1123–1130 (2023).
22. J Dauparas, et al., Robust deep learning–based protein sequence design using proteinmpnn. *Science* **378**, 49–56 (2022).
23. C Zhang, M Shine, AM Pyle, Y Zhang, US-align: universal structure alignments of proteins, nucleic acids, and macromolecular complexes. *Nat. Methods* **19**, 1109–1115 (2022).
24. Y Song, et al., Score-based generative modeling through stochastic differential equations (2021).
25. JL Watson, et al., Broadly applicable and accurate protein design by integrating structure prediction networks and diffusion generative models. *bioRxiv* (2022).
26. J Ho, et al., Video diffusion models (2022).
27. Y Du, et al., Reduce, reuse, recycle: Compositional generation with energy-based diffusion models and mcmc (2023).
28. J Besag, Comments on "representations of knowledge in complex systems" by u. grenander and mi miller (1994).
29. J Yim, et al., Se(3) diffusion model with application to protein backbone generation (2023).
30. BA Kenyon, Checking your pdb file for clashing atoms (2023).
31. A Leaver-Fay, et al., Rosetta3: An Object-Oriented Software Suite for the Simulation and Design of Macromolecules. *Methods enzymology* **487**, 545–574 (2011).
32. M McPartlon, J Xu, An end-to-end deep learning method for protein side-chain packing and inverse folding. *Proc. Natl. Acad. Sci.* **120**, e2216438120 (2023) Publisher: Proceedings of the National Academy of Sciences.
